# The relationship between cognitive functioning and psychopathology in patients with psychiatric disorders: a transdiagnostic network analysis

**DOI:** 10.1017/S0033291721001781

**Published:** 2023-01

**Authors:** UnYoung Chavez-Baldini, Dorien H. Nieman, Amos Keestra, Anja Lok, Roel J. T. Mocking, Pelle de Koning, Valeria V. Krzhizhanovskaya, Claudi L.H. Bockting, Geeske van Rooijen, Dirk J. A. Smit, Arjen L. Sutterland, Karin J. H. Verweij, Guido van Wingen, Johanna T.W. Wigman, Nienke C. Vulink, Damiaan Denys

**Affiliations:** 1 Department of Psychiatry, Amsterdam UMC, University of Amsterdam, Meibergdreef 9, Amsterdam, The Netherlands; 2 Informatics Institute, University of Amsterdam, Science Park 904, 1090, GH, Amsterdam, The Netherlands; 3 University Medical Center Groningen, University Center Psychiatry, Interdisciplinary Center Psychopathology and Emotion Regulation, University of Groningen, CC72, P.O. Box 30.001, 9700 RB, Groningen, The Netherlands

**Keywords:** transdiagnostic, cognitive function, psychopathology, network analysis

## Abstract

**Background:**

Patients with psychiatric disorders often experience cognitive dysfunction, but the precise relationship between cognitive deficits and psychopathology remains unclear. We investigated the relationships between domains of cognitive functioning and psychopathology in a transdiagnostic sample using a data-driven approach.

**Methods:**

Cross-sectional network analyses were conducted to investigate the relationships between domains of psychopathology and cognitive functioning and detect clusters in the network. This naturalistic transdiagnostic sample consists of 1016 psychiatric patients who have a variety of psychiatric diagnoses, such as depressive disorders, anxiety disorders, obsessive−compulsive and related disorders, and schizophrenia spectrum and other psychotic disorders. Psychopathology symptoms were assessed using various questionnaires. Core cognitive domains were assessed with a battery of automated tests.

**Results:**

Network analysis detected three clusters that we labelled: general psychopathology, substance use, and cognition. Depressive and anxiety symptoms, verbal memory, and visual attention were the most central nodes in the network. Most associations between cognitive functioning and symptoms were negative, i.e. increased symptom severity was associated with worse cognitive functioning. Cannabis use, (subclinical) psychotic experiences, and anhedonia had the strongest total negative relationships with cognitive variables.

**Conclusions:**

Cognitive functioning and psychopathology are independent but related dimensions, which interact in a transdiagnostic manner. Depression, anxiety, verbal memory, and visual attention are especially relevant in this network and can be considered independent transdiagnostic targets for research and treatment in psychiatry. Moreover, future research on cognitive functioning in psychopathology should take a transdiagnostic approach, focusing on symptom-specific interactions with cognitive domains rather than investigating cognitive functioning within diagnostic categories.

## Introduction

It is becoming increasingly accepted that psychiatric diagnostic categories are pragmatic, man-made constructs that should be interpreted as guidelines for clinical communication rather than true representations of underlying disorders (Marshall, 2020). Symptom expression is not disorder-specific, possibly explaining findings of high rates of comorbidity, general treatment effects, heterogeneity within disorders, and a lack of objective biomarkers (Hyman, 2010; Ofrat & Krueger, 2012; Olbert, Gala, & Tupler, 2014).

To remedy this, a transdiagnostic approach has been put forth to advance the understanding of psychopathology in which mental disorders are not deemed categorically distinct entities. This transdiagnostic approach acknowledges that expression of symptoms can manifest across disorders and takes into account other clinically relevant dimensions, including cognitive functioning. Consortiums and research initiatives, such as the Research Domain Criteria (RDoC) framework (Insel et al., 2010) and the Hierarchical Taxonomy of Psychopathology (HiTOP) consortium (Kotov et al., 2017), have been created with the goal to elucidate the nature of psychopathology without being bound to diagnostic categories. The Across study is among these research initiatives in its effort to employ a transdiagnostic approach to increase insight into the relationship among cognitive functioning, psychiatric symptoms, and biological parameters across psychiatric disorders (Nieman et al., 2020).

Previous research indicates that individuals with mental disorders often experience cognitive deficits (Iosifescu, 2012; Millan et al., 2012). For instance, executive dysfunction and memory deficits are apparent in patients with schizophrenia, depression, obsessive−compulsive disorder, post-traumatic stress disorder, and bipolar disorder (Czepielewski et al., 2015; Dere, Pause, & Pietrowsky, 2010; Snyder, Miyake, & Hankin, 2015). Cognitive dysfunction cuts across disorders and should therefore be considered a transdiagnostic dimension (East-Richard, R-Mercier, Nadeau, & Cellard, 2019; McTeague, Goodkind, & Etkin, 2016). Additionally, cognitive dysfunction associated with psychiatric disorders has been shown to persist into remission (Balanzá-Martínez et al., 2005; Iosifescu, 2012; Semkovska et al., 2019) and is predictive for recurrences (Ahern, Bockting, & Semkovska, 2019), suggesting that it is a construct partly independent from psychiatric symptoms. However, the psychopathological component of psychiatric disorders tends to garner the most attention while cognitive functioning remains neglected. Cognitive deficits, nonetheless, have been associated with psychosocial dysfunction in patients with schizophrenia (Shamsi et al., 2011), bipolar disorder (Depp et al., 2012), and depression (McIntyre et al., 2013). The transdiagnostic nature of cognitive dysfunction and its heavy impact on daily functioning makes it therefore an important target for treatment (Millan et al., 2012). Treating cognitive dysfunction in addition to psychopathology could lead to better outcomes for patients.

One statistical method ideally suited for transdiagnostic research is network analysis (Borsboom, 2017). Network analysis has been at the forefront of the broader paradigm shift in psychiatry as an alternative to the more traditional perspective of disorders as latent causes of symptoms. A network approach to psychopathology instead views disorders as constituted by symptoms which cause and interact directly with each other (Borsboom, 2017). By assessing the relationship between symptoms, network analysis can provide insights into which symptoms are more central, which symptoms cluster together, and which symptoms bridge different clusters (Blanken et al., 2018; Cramer, Waldorp, van der Maas, & Borsboom, 2010). This has resulted in network models showing how symptoms, such as psychotic and depressive symptoms (van Rooijen et al., 2017), interact with each other. Network analysis can also elucidate interactions of symptoms with other clinically relevant factors, such as anxiety with attentional bias (Heeren & McNally, 2016) and cognition with stress as assessed with cortisol levels (Hinkelmann et al., 2009). Although some network analyses on the relationship between cognitive functioning and psychopathology have been conducted (Galderisi et al., 2018; Heeren & McNally, 2016), there is still a lack of research using a transdiagnostic network approach combining cognitive functioning and psychopathology.

The present study aims to elucidate the relationship between domains of cognitive functioning (i.e. visual attention, executive function, verbal and episodic memory, and alertness) and psychiatric symptoms (e.g. depression and anxiety) using a transdiagnostic approach (i.e. across disorders). In a large naturalistic transdiagnostic sample of patients with psychiatric disorders, we aim to (1) conduct a network analysis with cognitive measures and psychiatric symptoms, (2) perform a centrality analysis to detect which variables are important within the network, and (3) detect clusters using exploratory graph analysis.

## Methods

### Sample

The naturalistic sample consists of 1016 psychiatric patients recruited during intakes at outpatient clinic of the Department of Psychiatry at the Amsterdam University Medical Centers (UMC), location Academic Medical Center (AMC), in Amsterdam, the Netherlands. Inclusion criteria were: age 14–75 years, ability to give informed consent, having a *DSM-IV-TR* or *DSM-V* diagnosis as determined by a trained psychiatrist, and being fluent in Dutch. Exclusion criteria were: acute high risk of suicide, unstable medical disorder, premorbid IQ < 70, history of seizure or clinically significant abnormality of the neurological system. Written informed consent was obtained from patients and their parents (if underage). Patients could discontinue participation from the study at any time.

### Procedure

The Across study is an ongoing, observational longitudinal cohort study and consists of the assessment of cognitive performance, psychiatric symptoms, and collection of biological data (DOI 10.17605/OSF.IO/YHVTB). The study instruments and procedures are described in Nieman et al. (2020). Patients underwent extensive psychiatric and medical assessments at the outpatient clinic, performed by experienced psychiatrists and psychologists, and were then invited to participate in the study. The current study used cross-sectional data on cognitive performance and psychiatric symptoms. Cognitive performance was assessed with a computerized battery, followed by self-report questionnaires about various symptoms that were filled out on a computer. Patients were not required to abstain from substance or medication use before participation and were able to participate at any point of their clinical trajectory (e.g. before, during, or after treatment), which could influence their cognitive functioning and/or symptomology. However, this is an observational, naturalistic study that aims to reflect the reality of patients regular functioning. The study protocol was approved by the Medical Ethical Review Committee of the Amsterdam UMC (ABR no. NL55751.018.15), and data are stored according European to privacy laws.

### Materials

Table 1 shows the instruments used to assess the different variables (nodes). For a detailed description of the instruments, see online Table S1 in the Supplementary Materials. To aid interpretability, cognitive variables were coded positively (higher is better) and psychopathology/substance use variables were coded negatively (higher is worse). All variables were continuous.

**Table 1.** Overview of measures

Domains and variables	Instruments	Nodes^a^
Cognitive functioning	Cambridge Neuropsychological Test Automated Battery (CANTAB; Cambridge Cognition, 2018; Sandberg, 2011)	CANTAB subtest scores: Choice Reaction Time (CRT), Intra/ Extradimensional Set Shift (IED), One Touch Stockings of Cambridge (OTS), Paired Associates Learning (PAL), Rapid Visual Processing (RVP), Spatial Working Memory (SWM), Verbal Recognition Memory (VRM)
	California Verbal Learning Test (CVLT; Delis, Kramer, Kaplan, & Ober, 2000)	CVLT total learning achievement
	Groninger Intelligence Test (GIT; Luteijn & Van der Ploeg, 1983)	GIT verbal fluency (category animals)
Intelligence	Dutch National Adult Reading Test (NART; Schmand, Bakker, Saan, & Louman, 1991)	Premorbid IQ score
Substance use	Alcohol Use Disorder Identification Test (AUDIT; Saunders, Aasland, Babor, De la Fuente, & Grant, 1993)^b^	AUDIT total score
	Cannabis Use Disorder Identification Test (CUDIT; Adamson & Sellman, 2003)^b^	CUDIT total score
Psychopathology	Prodromal Questionnaire-16 (PQ-16; Ising et al., 2012)	PQ-16 total score
	Yale-Brown Obsessive Compulsive Scale (Y-BOCS; Goodman et al., 1989)^b^	Y-BOCS total score
	Hamilton Anxiety Scale (HAM-A; Hamilton, Schutte, & Malouff, 1976)	HAM-A total score
	Inventory of Depressive Symptomology Self-Report (IDS-SR; Rush et al., 1986)	IDS-SR total score
	Impact of Events Scale- Revised (IES-R; Weiss & Marmar, 1997)^b^	IES-R total score
	Hedonism Scale (Rombouts & Van-Kuilenburg, 1988)^b^	Hedonism total score
	Self-esteem Rating Scale- Short Form (SERS-SF; Lecomte, Corbière, & Laisné, 2006)	SERS-SF total score
	Work and Social Adjustment Scale (WSAS; Mundt, Marks, Shear, & Greist, 2002)^b^	WSAS total score
	Social Interaction Anxiety Scale (SIAS; Mattick & Clarke, 1998)	SIAS total score

a The psychopathology and substance use nodes in this study were sum scores of symptom domains rather than individual symptoms.

b Assessed only for adult patients (18–75 years old) and put as missing in analyses for underage patients.

### Network analysis

The analyses consisted of network estimation and visualization, cluster detection, and centrality analysis performed in R Version 1.2.5042 (R Core Team, 2020). The network estimation procedure resulted in a set of relationships between variables that can be visualized in a network in which the variables are nodes that are connected by a set of edges representing the estimated relationships. Network estimation was conducted with the R-package *qgraph* (Epskamp, Cramer, Waldorp, Schmittmann, & Borsboom, 2012). The procedure started with a partial correlation network, in which correlations between two variables are corrected for all the other variables in the network. The extended Bayesian information criterion graphical least absolute shrinkage (EBICglasso) procedure (Chen & Chen, 2008; Epskamp & Fried, 2018; Foygel & Drton, 2010; Friedman, Hastie, & Tibshirani, 2008) was then applied to select edges by using a penalty, which decreases the strength of some of the parameter estimates, while others are set to zero. Following earlier network analyses, we used a *γ* of 0.5 for an optimal balance between density and sparseness (van Borkulo et al., 2015). This resulted in a sparse network, in which the absence of an edge is interpreted as the conditional independence of two nodes given other variables (Friedman et al., 2008).

Visualization of the network was performed with *qgraph* (Epskamp et al., 2012), using the Fruchterman−Reingold algorithm (Fruchterman & Reingold, 1991), which placed nodes that are more connected closer together and nodes which have higher centrality indices closer to the center of the graph. Stronger edges were depicted as thicker lines between nodes. Positive associations were depicted in green and negative associations in red. Due to the coding, we expected most edges between domains of cognitive functioning and psychopathology to be red whereas edges between psychopathology nodes and edges between cognitive nodes were expected to be green.

Analysis of the network stability (Epskamp, Borsboom, & Fried, 2018) was conducted (see online Appendix 1 in the Supplementary Materials) to give an indication of how reliable the estimated network is.

We computed the centrality index strength (Bringmann et al., 2019; Epskamp et al., 2018) using *qraph* (Epskamp et al., 2012). Strength is calculated as the total of all edges of that node and indicates the degree of association of that node to its neighbors and the relative importance of each node in the full network. A node that has high strength is a node that has many and/or strong connections to its neighbors, whereas lower strength indicates a node with fewer and/or weaker connections.

Cluster detection was conducted with an exploratory graph analysis (EGA) as implemented in the R-package *EGA* (Golino & Epskamp, 2017). EGA uses the ‘walktrap’ algorithm, which uses random walks to quantify the distance between any two nodes or clusters in a network (Pons & Latapy, 2006). Proximity between two nodes is defined as the degree of similarity of the distance of two nodes to the rest of the network. The walktrap algorithm then uses an agglomerative approach, grouping together the closest nodes or clusters step by step until all nodes are part of a cluster. The final step determines the point during the agglomeration in which the fraction of the strength of internal connections within the clusters compared to the external connections between clusters is optimized. This determines how the clusters within the network are finally defined. This step allowed us to identify subgroups of nodes that cluster together due to strong interconnectedness.

## Results

### Sample characteristics

Data of 1016 patients were included in the analyses. Overall, patients had an average premorbid IQ and a majority of the sample were of Caucasian ethnicity. On average, the sample consisted mostly of young and middle-aged adults and about half were male. The distribution of the primary diagnosis reflects the naturalistic patient population of the Amsterdam UMC, an expertise center for misophonia, early psychosis, anxiety and depressive disorders. Further results are presented in Table 2. Cognitive and psychopathology variable scores are shown in online Table S2 and CANTAB standard scores are shown in online Table S3 in the Supplementary Materials. Differences in cognitive and psychopathology variable scores per medication category are shown in online Table S4 in the Supplementary Materials. Except for alcohol use, cognitive flexibility, and alertness, there were significant differences in scores over types of medication, suggesting a possible influence of medication on cognitive and psychopathology variables. Generally, antidepressants, benzodiazepines, and sleep medication differed significantly and were related to worse symptomology. Furthermore, antipsychotic medication differed significantly and was related to worse cognitive function.

**Table 2.** Demographic and clinical characteristics of participants

Measure	Values (*N* = 1016)
Age (years), mean (S.D.)	34.7 (14.2)
Gender, male, no. (%)	478 (47.1)
Education, no. (%)	
Higher education	340 (33.5)
Vocational education	205 (20.2)
Secondary education	345 (40.0)
Primary education	47 (4.6)
No education	32 (3.1)
Unknown	47 (4.6)
Premorbid IQ (NART), mean (s.d.)	100.09 (13.3)
Ethnicity, Caucasian, No. (%)	878 (86.5)
Marital status, married, no. (%)	316 (31.1)
DSM diagnostic category^a^, no. (%)	
Schizophrenia spectrum and other psychotic disorders	185 (18.2)
Depressive disorder	111 (10.9)
Anxiety disorder	60 (5.9)
Obsessive−compulsive and related disorders	177 (17.5)
Impulse-control disorder NOS (misophonia)	353 (34.7)
Other disorders^b^	130 (12.8)
Medication use, no. (%)	
Antidepressants	213 (21.0)
Antipsychotics	169 (16.6)
Benzodiazepines	30 (3.0)
Sleep medication	15 (1.5)
Mood stabilizers	8 (0.8)
Psychostimulants	8 (0.8)
Other	168 (16.5)
None	392 (38.6)
Unknown	13 (1.3)

NART, Dutch National Adult Reading Test; DSM, Diagnostic and Statistical Manual of Mental Disorders; NOS, not otherwise specified.

a Diagnostic category is only for the primary diagnosis.

b Other disorder category includes: substance use disorders (*n* = 20), eating disorders (*n* = 5), neurodevelopmental disorders (*n* = 29), sexual disorders (*n* = 2), sleep disorders (*n* = 2), dissociative disorders (*n* = 1), adjustment disorders (*n* = 4), bipolar disorders (*n* = 28), and personality disorders (*n* = 12).

### Network analysis

**Fig. 1.**
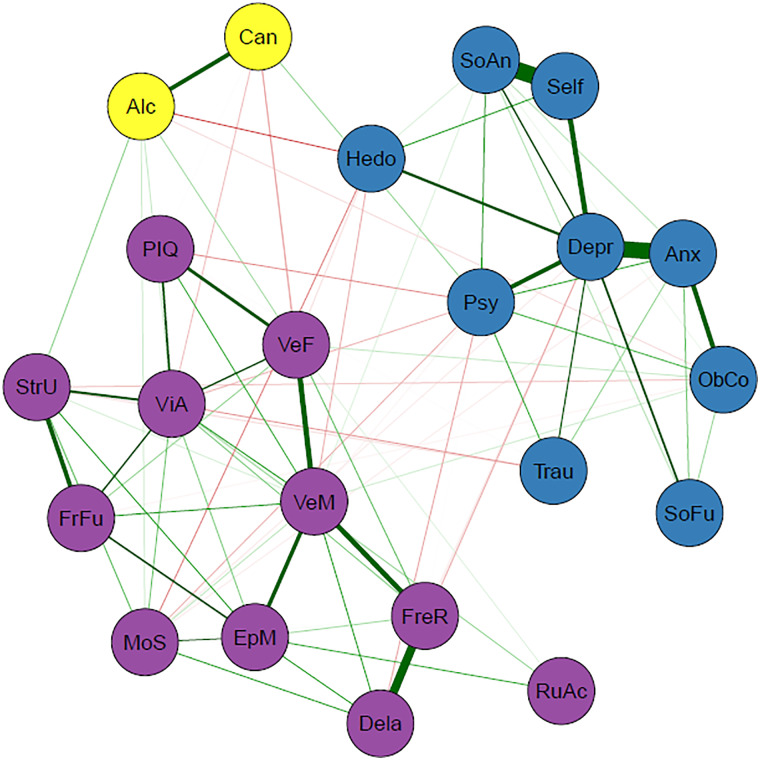
Transdiagnostic network of cognitive and psychopathology domains (*N* = 1016). Nodes represent the variables included in the network and edges indicate an association between two nodes. Green edges represent positive associations whereas red edges represent negative associations, and thickness of an edge represents the strength of association between two nodes. The color of each node indicates to which cluster it belongs according to the EGA: cognition (purple), general psychopathology (blue), or substance use (yellow). Psychopathology domains: Alc = alcohol (ab)use (AUDIT); Anx = anxiety symptoms (HAM-A); Can = cannabis (ab)use (CUDIT); Depr = depressive symptoms (IDS); Hed = hedonism questionnaire; ObCo = obsessive−compulsive symptoms (Y-BOCS); Psy = (subclinical) psychotic experiences (PQ-16); Self = self-esteem (SERS-SF); SoAn = social anxiety symptoms (SIAS); SoFu = poor psychosocial functioning (WSAS); Trau = post-traumatic stress symptoms (IES-R). Cognitive domains: Dela = verbal recognition memory-delayed (VRM); EpM = episodic memory and learning (PAL); FreR = verbal recognition memory-immediate (VRM); FrFu = planning test (OTS); MoS = alertness and motor speed (CRT); PIQ = premorbid IQ (NART); RuAc = rule acquisition and attentional set shifting, cognitive flexibility (IED); StrU = strategy use (SWM); VeF = verbal fluency (GIT); VeM = verbal memory (CVLT); ViA = sustained visual attention and processing speed (RVP).

The network of the cognitive functioning and psychopathology domains is visualized in Fig. 1. Three clusters were detected, indicated by different colors in the figure which were labelled: general psychopathology symptoms (blue), substance use (yellow), and cognition (purple). A weights matrix of the network can be seen in online Table S5 in the Supplementary Materials. The stability analysis revealed that most of the edges in the network were stable, indicating that the estimated network was robust (online Fig. S4 in the Supplementary Materials). Variables with the highest strength were depression, anxiety, verbal memory, and visual attention.

As expected, within-cluster edges between psychopathology and within-cluster edges between cognitive domain were mostly positive. Cross-cluster edges between cognitive domains and psychopathology contained mostly negative edges, in which increased symptom severity was associated with worse cognitive functioning. The total strengths of edges within clusters are notably higher than the total strength of edges between clusters, which is in line with our expectations because nodes cluster together due to strong interconnectedness. An overview of the edges in the network can be seen in Table 3. Closer inspection of the edges between cognitive domains and psychopathology showed that cognitive nodes have both positive and negative associations with different psychopathology nodes, whereas psychopathology nodes have either positive or negative associations with different cognitive nodes (online Table S6 in the Supplementary Materials). The obsessive−compulsive symptoms node was the only exception. Furthermore, (subclinical) psychotic experiences, cannabis use, and anhedonia had the strongest total negative relationships with cognitive variables while alcohol use had the strongest total positive relationship with cognitive variables (online Table S6 in the Supplementary Materials).

**Table 3.** Overview of the edges in the network^a^

	Total	Positive, no. (%)	Negative, no. (%)	Total strength	Positive strength	Negative strength
All edges	76	50 (66)	26 (34)	4.79	5.61	−0.82
Between psychopathology nodes^b^	25	23 (92)	2 (8)	3.16	3.27	−0.10
Between cognitive nodes	32	32 (100)	0 (0)	3.49	3.49	0.00
Psychopathology−cognition interaction	29	7 (24)	22 (76)	−0.33	0.19	−0.52

a First three columns are the number of edges, whereas the last three columns are the sums of the strengths of the edges. Rows indicate the set of edges under consideration.

b Substance use nodes are included.

To further assess the stability of the cluster detection in our analyses, we performed a bootstrap analysis of the EGA procedure for all networks, including the control networks. This analysis revealed that amongst all the networks, the original network exhibited the most stable cluster structure (online Table S7 in the Supplementary Materials). Stability checks also demonstrated that networks were stable despite changes in sample size, indicating that sample size did not largely influence this (online Fig. S5 in the Supplementary Materials).

Lastly, further network analyses were conducted as controls: one using binarized data to control for differences in measurement methods and scales and others to account for sample variation in the variables based on diagnostic category, age, sex, education level, and medication use (online Appendices 1 and 2 in the Supplementary Materials). All the control networks reproduced the original three-cluster network, except for one diagnostic network (without depressive disorders) and the without medication network, which produced four clusters. In both of these networks (without depressive disorders and without medication), the psychopathology cluster was split into two separate clusters. The separation of cognitive and psychopathological variables remained in all control networks (online Figs S6–S14 in the Supplementary Materials).

## Discussion

The present study used a network approach to investigate how domains of cognitive functioning and psychopathology cluster interact in a large transdiagnostic sample of patients with psychiatric disorders. This resulted in a fully connected network, showing that the domains are all closely associated. Cluster analysis detected three clusters in the network, labelled: cognition, general psychopathology, and substance use.

Cognitive functioning and psychopathology each form their own clusters, indicating that they are independent but related dimensions. The edges between the cognition and psychopathology clusters were relatively weak, which emphasizes their independence and further supports that cognitive function should be investigated in addition to and separately from psychiatric symptoms. Nonetheless, any associations, even if weak, may still be an important part of the etiology. The separation of these clusters also held across the control networks that were conducted to check the possible influence of diagnostic categories, suggesting that this interaction between cognitive domains and psychopathology domains is transdiagnostic and does not adhere to traditional diagnostic boundaries.

The formation of the separate clusters is in line with the hot−cold cognitive model in depression (Ahern et al., 2019; De Raedt & Koster, 2010; Roiser & Sahakian, 2013), in which ‘cold’ cognition is information processing without emotional influence (e.g. attentional control) and ‘hot’ cognition is information processing with emotional influence (e.g. mood-congruent attentional bias). The hot−cold cognitive model posits that non-affective cold cognitive dysfunctions contributes to the development of hot cognition (e.g. negative/catastrophic thoughts/beliefs), subsequently leading to psychiatric symptoms and further exacerbating cold cognitive dysfunctions. While we did not specifically measure hot cognition, it could be theorized that the psychiatric symptoms in our sample could also arise from cold cognitive deficits through hot cognition. Future research could investigate whether hot cognition acts as a bridge between cold cognition and symptoms. This model could be extended to other disorders as cognitive−affective processes are transdiagnostic (Iosifescu, 2012; Mansell, Harvey, Watkins, & Shafran, 2008). Although temporal relationships cannot be assessed in the current study, this also supports our result regarding the association between worse cognitive functioning and higher symptom severity.

Another possibility for the separate clusters is that cognitive functioning and psychopathology follow different clinical trajectories, as suggested by findings of premorbid and persisting cognitive deficits despite symptom remission (Allott, Fisher, Amminger, Goodall, & Hetrick, 2016; Balanzá-Martínez et al., 2005; Caspi et al., 2003; Semkovska et al., 2019). This could further tie in with the hot−cold cognitive model, in which premorbid cognitive deficits lead to psychiatric symptoms, and explain how cognitive functioning and psychopathology are related despite different trajectories.

Within the general psychopathology cluster, self-esteem, depressed mood, and (social) anxiety were strongly related. Low self-esteem can be considered a general transdiagnostic risk and maintenance factor in psychopathology (Zeigler-Hill, 2011) and is often related to depression and anxiety (Sowislo & Orth, 2013). The depressive symptoms node had the highest number of edges and highest total edge strength within the psychopathology cluster, meaning that it had the most relationships with other psychopathology domains. Depressive symptoms are often reported in patients with a variety of other disorders, such as subclinical psychosis, bipolar disorder, and obsessive−compulsive disorders (Fusar-Poli, Nelson, Valmaggia, Yung, & McGuire, 2012; Goldberg & Fawcett, 2012; Quarantini et al., 2011). Depressive symptoms could be considered a normal response to living with a psychiatric disorder, which can induce lowered self-esteem (Silverstone & Salsali, 2003), (self)stigma, and reduced possibilities in life. Successful treatment of the psychiatric disorder may subsequently reduce the depressive symptoms. There is also the possibility of bidirectional relationships between depressive symptoms and other clinical factors; however, this cannot be inferred with the current undirected network.

The substance use cluster is composed of the AUDIT and CUDIT. The formation of this cluster may have been influenced by the fact that the scales measure closely related constructs and are highly similar in form (i.e. the CUDIT was developed from the AUDIT) and/or because of the low prevalence of cannabis and alcohol use (i.e. a high number of individuals scored zeros on both scales). These possibilities were controlled for and the results led us to conclude that the original three clusters represent the most reliable clusters. This suggests substance use is related to the general psychopathology cluster but exhibits a certain independence at the same time. The general psychopathology cluster mostly contains affective symptoms, and while affective symptoms and substance (ab)use are related (Boden & Fergusson, 2011; Kedzior & Laeber, 2014), they are nonetheless different psychometrically and conceptually (emotions *v.* behavior) and fall under different spectra (internalizing *v.* externalizing; Kotov et al., 2017).

Within the cognition cluster, the verbal memory node had the highest number of edges and the highest total edge strength, indicating that verbal memory was related to many other cognitive domains. Verbal memory had the strongest association with verbal fluency. Interestingly, verbal memory has been shown to be predictive of remission for at-risk mental states individuals (Simon et al., 2012) and treatment response for PTSD (Scott et al., 2017) and comorbid depression−anxiety (Braund, Tillman, Palmer, & Harris, 2020). This may suggest that it could be beneficial to improve verbal memory deficits in patients with psychiatric disorders to improve treatment response and increase the chance of remission. Many therapies are verbal and verbal memory deficits may hamper therapeutic success because patients have more difficulty remembering the content of their therapy sessions.

Depression, anxiety, verbal memory, and visual attention have the highest strength in the network, implying that they play a central role in psychiatric disorders. These nodes have a strong influence on other nodes, and could have a significant impact on overarching psychopathology. A common factor here may be stress, which plays a role in the development and maintenance of many disorders (Conway, Raposa, Hammen, & Brennan, 2018; Nolen-Hoeksema & Watkins, 2011). Dysregulation of the central stress response system, the hypothalamic−pituitary−adrenal (HPA) axis, can lead to memory deficits (Wingenfeld & Wolf, 2014). Stress is also implicated in the etiology of emotional disturbances (McLaughlin, Conron, Koenen, & Gilman, 2010; Pechtel & Pizzagalli, 2011), including excess of negative emotion and distress, explaining the relative importance of depression and anxiety symptoms, which are often observed across psychiatric disorders (Kring, 2008).

Attentional deficits are also evident in patients with psychiatric disorders transdiagnostically (Millan et al., 2012). In the network, visual attention displays the strongest associations with strategy use (working memory), verbal fluency, and planning ability, which is in line with the executive-attention framework (Buckholtz & Meyer-Lindenberg, 2012; Millan et al., 2012). Previous studies have shown that dorsolateral prefrontal−cingulate−parietal network underpins the executive-attention framework (Buckholtz & Meyer-Lindenberg, 2012; Millan et al., 2012). Atypical connectivity within this network is often found across disorders, which may contribute to deficits in working memory, attention, and cognitive control (Buckholtz & Meyer-Lindenberg, 2012). Furthermore, associations of the visual cortex with the frontoparietal and default mode networks have been implicated in information processing (e.g. attention; Chadick & Gazzaley, 2011). In addition, hyperconnectivity between visual association cortex and both frontoparietal and default mode networks has been associated with a general liability for mental illness (i.e. *p*-factor; Elliott, Romer, Knodt, & Hariri, 2018). This could provide an underlying mechanism for the shared cognitive deficits that are present across disorders.

Interestingly, we found that nodes representing psychopathology tended to have consistent relationships with cognitive domains, while nodes representing cognitive domains had mixed relationships with psychopathology. For instance, depression was consistently related to worse functioning across cognitive domains. However, verbal memory functioning had different relationships to certain psychopathology domains: (subclinical) psychotic experiences were related to worse verbal memory, whereas obsessions and compulsions were related to better verbal memory. This encourages future research to focus on symptom-specific interactions with different cognitive domains, rather than investigating cognitive functioning within diagnostic categories. Research within diagnostic categories could also explain why clear findings on cognitive functioning in psychiatric diagnostic categories are hard to come by: specific symptoms and cognitive deficits may vary considerably within one diagnostic category, and patterns could emerge more clearly by investigating relationships between cognitive functioning and symptoms directly, rather than comparing them across categories.

Cannabis use, (subclinical) psychotic experiences, and anhedonia had the strongest overall negative association with cognitive functioning. Both (subclinical) psychotic-like experiences and cannabis use have been associated with cognitive dysfunction (Lindgren et al., 2010; Volkow et al., 2016). The relation between anhedonia and worse cognitive functioning, specifically motor speed, verbal memory, and fluency, may be partly explained by decreased connectivity within reward-related brain regions (Felger et al., 2015). Interestingly, psychosocial functioning was included in the symptom cluster and demonstrated no associations with cognitive functioning. This is somewhat unexpected because psychosocial functioning tends to be more strongly associated with cognitive functioning than with psychiatric symptoms (Depp et al., 2012; McIntyre et al., 2013; Shamsi et al., 2011), although associations with psychopathology, such as depression (Fried & Nesse, 2014) have been reported.

The main strengths of the current analysis are the inclusion of various cognitive and psychopathology domains and the large transdiagnostic sample. This means that the results may be generalizable to patients with a wide range of psychiatric disorders. The main limitation of the current study is the cross-sectional design, meaning that neither causal interactions nor the direction of relationships can be assessed. Using cross-sectional data is a problem in most network papers published so far, and the results must be interpreted with caution (Guloksuz, Pries, & Van Os, 2017). However, cross-sectional networks could still give insight into the co-occurrence of symptoms (Bos et al., 2017). The Across study is ongoing; hence, future analyses will entail longitudinal data and biological markers. Another limitation is that sum scores of questionnaires were used. This was done to reduce the number of nodes in the network at the expense of losing information about relationships between individual items. Questionnaires also had differences in scoring and scaling properties, which could have influenced the pattern of edges and clusters detected. Most questionnaires were also self-reported, introducing a possible interpretation and self-report bias. However, the self-report questionnaires that were used are psychometrically valid and provide important information from the patients’ perspective. Additionally, due to the naturalistic character of our sample and the fact that our department does not specialize in treatment for these patient groups, some diagnostic groups were relatively underrepresented in our sample (e.g. substance use and neurodevelopmental disorders) and were therefore grouped together in an ‘Other disorders’ category.

Furthermore, the lack of a healthy control group is a limitation in the paper. Although we attempt to use a dimensional approach when assessing the psychiatric domains included in this study, we cannot capture the entire continuum without a healthy control or general population group. Due to a lack of a well-matched healthy control group, it is not clear to what extent cognitive deficits are present and whether these deficits are clinically relevant. A further limitation is that the network was not adjusted for medication use, which can impact cognitive function. This is, however, a naturalistic study of patients with psychiatric disorders, reflecting clinical reality of medication use. We would also like to note that there is probably an interaction effect between the disorder, clinical severity, and medication use, which would make it difficult to disentangle the effect of medication and the effect of the disorder on cognitive functioning.

Moreover, while the use of a transdiagnostic approach is a strength, we recognize the potential shortcoming of not analyzing specific relationships across diagnostic categories. However, control networks with diagnostic categories removed demonstrated that the structure of network is fairly robust to potential differences between subgroups. Finally, labelling of clusters is, as always with such techniques, rather subjective. Altogether, this study should be seen as a first investigation of the structure of the relationship between psychopathology and cognitive functioning. Future exploration can focus on parts of the network to investigate how specific items relate to each other and to cognitive measures.

The results of this study support the notion that cognitive functioning and psychopathology are independent but related dimensions, which interact in a transdiagnostic manner. Thus, it cannot be assumed that treating symptoms will alleviate cognitive deficits, and future studies should specifically assess if typical treatments influence cognitive dysfunction. Cognitive deficits, however, are usually undertreated, suggesting a need for treatments specifically targeting cognitive dysfunction in patients with psychiatric disorders, regardless of diagnosis. Interventions for cognitive dysfunction, such as cognitive remediation, which tend to be heavily geared towards patients with schizophrenia or bipolar disorders, should perhaps be considered transdiagnostic. Furthermore, depression, anxiety, verbal memory, and visual attention seem to play central roles across disorders and should therefore be the focus of transdiagnostic research and treatment.

## Supplementary material

For supplementary material accompanying this paper visit https://doi.org/10.1017/S0033291721001781. click here to view supplementary material

## References

[ref1] Adamson, S. J., & Sellman, J. D. (2003). A prototype screening instrument for cannabis use disorder: The Cannabis Use Disorders Identification Test (CUDIT) in an alcohol-dependent clinical sample. Drug and Alcohol Review, 22(3), 309–315. 10.1080/0959523031000154454. 15385225

[ref2] Ahern, E., Bockting, C. L., & Semkovska, M. (2019). A hot-cold cognitive model of depression: Integrating the neuropsychological approach into the cognitive theory framework. Clinical Psychology in Europe, 1, e34396. 10.32872/cpe.v1i3.34396.

[ref3] Allott, K., Fisher, C. A., Amminger, G. P., Goodall, J., & Hetrick, S. (2016). Characterizing neurocognitive impairment in young people with major depression: State, trait, or scar? Brain and Behavior, 6(10), e00527. 10.1002/brb3.527. 27781141 PMC5064339

[ref4] Balanzá-Martínez, V., Tabarés-Seisdedos, R., Selva-Vera, G., Martínez-Arán, A., Torrent, C., Salazar-Fraile, J., … Gómez-Beneyto, M. (2005). Persistent cognitive dysfunctions in bipolar I disorder and schizophrenic patients: A 3-year follow-up study. Psychotherapy and Psychosomatics, 74(2), 113–119. 10.1159/000083170. 15741761

[ref5] Blanken, T. F., Deserno, M. K., Dalege, J., Borsboom, D., Blanken, P., Kerkhof, G. A., & Cramer, A. O. (2018). The role of stabilizing and communicating symptoms given overlapping communities in psychopathology networks. Scientific Reports, 8(1), 5854. 10.1038/s41598-018-24224-2. 29643399 PMC5895626

[ref6] Boden, J. M., & Fergusson, D. M. (2011). Alcohol and depression. Addiction, 106(5), 906–914. 10.1111/j.1360-0443.2010.03351.x. 21382111

[ref7] Borsboom, D. (2017). A network theory of mental disorders. World Psychiatry, 16(1), 5–13. 10.1002/wps.20375. 28127906 PMC5269502

[ref8] Bos, F. M., Snippe, E., de Vos, S., Hartmann, J. A., Simons, C. J. P., van der Krieke, L., … Wichers, M. (2017). Can we jump from cross-sectional to dynamic? Interpretations of networks implications for the network perspective in psychiatry. Psychotherapy and Psychosomatics, 86(3), 175–177. doi: 10.1159/000453583. 28490028 PMC5516409

[ref9] Braund, T. A., Tillman, G., Palmer, D. M., & Harris, A. W. F. (2020). Verbal memory predicts treatment outcome in syndromal anxious depression: An iSPOT-D report. Journal of Affective Disorders, 260, 245–253. 10.1016/j.jad.2019.09.028. 31513968

[ref10] Bringmann, L. F., Elmer, T., Epskamp, S., Krause, R. W., Schoch, D., Wichers, M., … Snippe, E. (2019). What do centrality measures measure in psychological networks? The Journal of Abnormal Psychology, 128(8), 892–903. 10.3929/ethz-b-000368126. 31318245

[ref11] Buckholtz, J. W., & Meyer-Lindenberg, A. (2012). Psychopathology and the human connectome: Toward a transdiagnostic model of risk for mental illness. Neuron, 74(6), 990–1004. 10.1016/j.neuron.2012.06.002. 22726830

[ref12] Cambridge Cognition (2018). CANTAB® [Cognitive assessment software]. Cambridge Cognition Ltd. Available at www.cantab.com.

[ref13] Caspi, A., Reichenberg, A., Weiser, M., Rabinowitz, J., Kaplan, Z. E., Knobler, H., … Davidson, M. (2003). Cognitive performance in schizophrenia patients assessed before and following the first psychotic episode. Schizophrenia Research, 65(2), 87–94. 10.1016/S0920-9964(03)00056-2. 14630301

[ref14] Chadick, J. Z., & Gazzaley, A. (2011). Differential coupling of visual cortex with default or frontal-parietal network based on goals. Nature Neuroscience, 14(7), 830–832. doi:10.1038/nn.2823. 21623362 PMC3125492

[ref15] Chen, J., & Chen, Z. (2008). Extended Bayesian information criteria for model selection with large model spaces. Biometrika, 95(3), 759–771. 10.1093/biomet/asn034.

[ref16] Conway, C. C., Raposa, E. B., Hammen, C., & Brennan, P. A. (2018). Transdiagnostic pathways from early social stress to psychopathology: A 20-year prospective study. Journal of Child Psychology and Psychiatry, 59(8), 855–862. doi:10.1111/jcpp.12862. 29315560 PMC12490286

[ref17] Cramer, A. O. J., Waldorp, L. J., van der Maas, H. L. J., & Borsboom, D. (2010). Comorbidity: A network perspective. Behavioral and Brain Sciences, 33(2–3), 137–150. doi:10.1017/S0140525X09991567. 20584369

[ref18] Czepielewski, L. S., Massuda, R., Goi, P., Sulzbach-Vianna, M., Reckziegel, R., Costanzi, M., … Gama, C. S. (2015). Verbal episodic memory along the course of schizophrenia and bipolar disorder: A new perspective. European Neuropsychopharmacology, 25(2), 169–175. 10.1016/j.euroneuro.2014.09.006. 25311898

[ref19] Delis, D. C., Kramer, J., Kaplan, E., & Ober, B. A. (2000). CVLT-II: California verbal learning test: Adult version. Cleaveland, Ohio: The Psychological Corporation.

[ref20] Depp, C. A., Mausbach, B. T., Harmell, A. L., Savla, G. N., Bowie, C. R., Harvey, P. D., & Patterson, T. L. (2012). Meta-analysis of the association between cognitive abilities and everyday functioning in bipolar disorder. Bipolar Disorders, 14(3), 217–226. 10.1111/j.1399-5618.2012.01011.x. 22548895 PMC3396289

[ref21] De Raedt, R., & Koster, E. H. W. (2010). Understanding vulnerability for depression from a cognitive neuroscience perspective: A reappraisal of attentional factors and a new conceptual framework. Cognitive, Affective, & Behavioral Neuroscience, 10(1), 50–70. doi:10.3758/CABN.10.1.50. 20233955

[ref22] Dere, E., Pause, B. M., & Pietrowsky, R. (2010). Emotion and episodic memory in neuropsychiatric disorders. Behavioural Brain Research, 215(2), 162–171. 10.1016/j.bbr.2010.03.017. 20227444

[ref23] East-Richard, C., R-Mercier, A., Nadeau, D., & Cellard, C. (2019). Transdiagnostic neurocognitive deficits in psychiatry: A review of meta-analyses. Canadian Psychology/Psychologie canadienne, 61(3), 194–214.

[ref24] Elliott, M. L., Romer, A., Knodt, A. R., & Hariri, A. R. (2018). A connectome-wide functional signature of transdiagnostic risk for mental illness. Biological Psychiatry, 84(6), 452–459. 29779670 10.1016/j.biopsych.2018.03.012 PMC6119080

[ref25] Epskamp, S., Borsboom, D., & Fried, E. I. (2018). Estimating psychological networks and their accuracy: A tutorial paper. Behavior Research Methods, 50(1), 195–212. 10.3758/s13428-017-0862-1. 28342071 PMC5809547

[ref26] Epskamp, S., Cramer, A. O., Waldorp, L. J., Schmittmann, V. D., & Borsboom, D. (2012). qgraph: Network visualizations of relationships in psychometric data. Journal of Statistical Software, 48(4), 1–18. https://hdl.handle.net/11245/1.380173.

[ref27] Epskamp, S., & Fried, E. I. (2018). A tutorial on regularized partial correlation networks. Psychological Methods, 23(4), 617–634. https://psycnet.apa.org/doi/10.1037/met0000167. 29595293 10.1037/met0000167

[ref28] Felger, J. C., Li, Z., Haroon, E., Woolwine, B. J., Jung, M. Y., Hu, X., & Miller, A. H. (2015). Inflammation is associated with decreased functional connectivity within corticostriatal reward circuitry in depression. Molecular Psychiatry, 21, 1358. doi:10.1038/mp.2015.168. 26552591 PMC4862934

[ref29] Foygel, R., & Drton, M. (2010). Extended Bayesian information criteria for Gaussian graphical models. Paper presented at the Advances in Neural Information Processing Systems.

[ref30] Fried, E. I., & Nesse, R. M. (2014). The impact of individual depressive symptoms on impairment of psychosocial functioning. PLoS One, 9(2). https://dx.doi.org/10.1371%2Fjournal.pone.0090311. 10.1371/journal.pone.0090311 PMC3938686 24587318

[ref31] Friedman, J., Hastie, T., & Tibshirani, R. (2008). Sparse inverse covariance estimation with the graphical lasso. Biostatistics (Oxford, England), 9(3), 432–441. 10.1093/biostatistics/kxm045. 18079126 PMC3019769

[ref32] Fruchterman, T. M., & Reingold, E. M. (1991). Graph drawing by force-directed placement. Software: Practice and Experience, 21(11), 1129–1164. 10.1002/spe.4380211102.

[ref33] Fusar-Poli, P., Nelson, B., Valmaggia, L., Yung, A. R., & McGuire, P. K. (2012). Comorbid depressive and anxiety disorders in 509 individuals with an at-risk mental state: Impact on psychopathology and transition to psychosis. Schizophrenia Bulletin, 40(1), 120–131. doi:10.1093/schbul/sbs136 %J Schizophrenia Bulletin. 23180756 PMC3885287

[ref34] Galderisi, S., Rucci, P., Kirkpatrick, B., Mucci, A., Gibertoni, D., Rocca, P., … Aguglia, E. (2018). Interplay among psychopathologic variables, personal resources, context-related factors, and real-life functioning in individuals with schizophrenia: A network analysis. JAMA Psychiatry, 75(4), 396–404. doi: 10.1001/jamapsychiatry.2017.4607. 29450447 PMC5875306

[ref35] Goldberg, D., & Fawcett, J. (2012). The importance of anxiety in both major depression and bipolar disorder. Depression and Anxiety, 29(6), 471–478. doi: 10.1002/da.21939. 22553107

[ref36] Golino, H. F., & Epskamp, S. (2017). Exploratory graph analysis: A new approach for estimating the number of dimensions in psychological research. PLoS One, 12(6), e0174035. doi: 10.1371/journal.pone.0174035. 28594839 PMC5465941

[ref37] Goodman, W., Price, L., Rasmussen, S., Mazure, C., Fleischmann, R., Hill, C., … Charney, D. (1989). Yale-brown obsessive compulsive scale (Y-BOCS): I. Development, use, and reliability. Archives of General Psychiatry, 46, 1006–1011. doi:10.1001/archpsyc.1989.01810110048007. 2684084

[ref38] Guloksuz, S., Pries, L., & Van Os, J. (2017). Application of network methods for understanding mental disorders: Pitfalls and promise. Psychological Medicine, 47(16), 2743–2752. 10.1017/S0033291717001350. 28578740

[ref39] Hamilton, M., Schutte, N., & Malouff, J. (1976). Hamilton anxiety scale (HAMA). In N. S. Schutte, & J. M. Malouff (Eds.), Sourcebook of Adult Assessment: Applied Clinical Psychology (pp. 154–157). New York: Plenum Press.

[ref40] Heeren, A., & McNally, R. J. (2016). An integrative network approach to social anxiety disorder: The complex dynamic interplay among attentional bias for threat, attentional control, and symptoms. Journal of Anxiety Disorders, 42, 95–104. 10.1016/j.janxdis.2016.06.009. 27395806

[ref41] Hinkelmann, K., Moritz, S., Botzenhardt, J., Riedesel, K., Wiedemann, K., Kellner, M., & Otte, C. (2009). Cognitive impairment in major depression: Association with salivary cortisol. Biological Psychiatry, 66(9), 879–885. 10.1016/j.biopsych.2009.06.023. 19709646

[ref42] Hyman, S. E. (2010). The diagnosis of mental disorders: The problem of reification. Annual Review of Clinical Psychology, 6, 155–179. 10.1146/annurev.clinpsy.3.022806.091532. 17716032

[ref43] Insel, T., Cuthbert, B., Garvey, M., Heinssen, R., Pine, D. S., Quinn, K., … Wang, P. (2010). Research domain criteria (RDoC): Toward a new classification framework for research on mental disorders. American Journal of Psychiatry, 167(7), 748–751. 10.1176/appi.ajp.2010.09091379. 20595427

[ref44] Iosifescu, D. V. (2012). The relation between mood, cognition and psychosocial functioning in psychiatric disorders. European Neuropsychopharmacology, 22, S499–S504. 10.1016/j.euroneuro.2012.08.002. 22959115

[ref45] Ising, H. K., Veling, W., Loewy, R. L., Rietveld, M. W., Rietdijk, J., Dragt, S., … Linszen, D. H. (2012). The validity of the 16-item version of the Prodromal Questionnaire (PQ-16) to screen for ultra high risk of developing psychosis in the general help-seeking population. Schizophrenia Bulletin, 38(6), 1288–1296. 10.1093/schbul/sbs068. 22516147 PMC3713086

[ref46] Kedzior, K. K., & Laeber, L. T. (2014). A positive association between anxiety disorders and cannabis use or cannabis use disorders in the general population − A meta-analysis of 31 studies. BMC Psychiatry, 14(1), 136. doi:10.1186/1471-244X-14-136. 24884989 PMC4032500

[ref47] Kotov, R., Krueger, R. F., Watson, D., Achenbach, T. M., Althoff, R. R., Bagby, R. M., … Clark, L. A. (2017). The hierarchical taxonomy of psychopathology (HiTOP): A dimensional alternative to traditional nosologies. Journal of Abnormal Psychology, 126(4), 454. https://psycnet.apa.org/doi/10.1037/abn0000258. 28333488 10.1037/abn0000258

[ref48] Kring, A. M.. (2008). Emotion disturbances as transdiagnostic processes in psychopathology. In M. Lewis, J.M. Haviland-Jones, & L.F. Barret (Eds.), Handbook of Emotions (3, pp. 691–705). New York, New York: The Guildford Press.

[ref49] Lecomte, T., Corbière, M., & Laisné, F. (2006). Investigating self-esteem in individuals with schizophrenia: Relevance of the self-esteem rating scale-short form. Psychiatry Research, 143(1), 99–108. 10.1016/j.psychres.2005.08.019. 16725210

[ref50] Lindgren, M., Manninen, M., Laajasalo, T., Mustonen, U., Kalska, H., Suvisaari, J., … Therman, S. (2010). The relationship between psychotic-like symptoms and neurocognitive performance in a general adolescent psychiatric sample. Schizophrenia Research, 123(1), 77–85. 10.1016/j.schres.2010.07.025. 20729039

[ref51] Luteijn, F., & Van der Ploeg, F. A. (1983). Groninger intelligentie test: Handleiding [Groninger intelligence test: Manual]. Lisse: Swets, Zeitlinger BV.

[ref52] Mansell, W., Harvey, A., Watkins, E. R., & Shafran, R. (2008). Cognitive behavioral processes across psychological disorders: A review of the utility and validity of the transdiagnostic approach. International Journal of Cognitive Therapy, 1(3), 181–191. doi:10.1521/ijct.2008.1.3.181.

[ref53] Marshall, M. (2020). The hidden links between mental disorders. Nature, 581, 19–21. doi:10.1038/d41586-020-00922-8. 32372044

[ref54] Mattick, R. P., & Clarke, J. C. (1998). Development and validation of measures of social phobia scrutiny fear and social interaction anxiety. Behaviour Research and Therapy, 36(4), 455–470. 9670605 10.1016/s0005-7967(97)10031-6

[ref55] McIntyre, R. S., Cha, D. S., Soczynska, J. K., Woldeyohannes, H. O., Gallaugher, L. A., Kudlow, P., … Baskaran, A. (2013). Cognitive deficits and functional outcomes in major depressive disorder: Determinants, substrates, and treatment interventions. Depression and Anxiety, 30(6), 515–527. 10.1002/da.22063. 23468126

[ref56] McLaughlin, K. A., Conron, K. J., Koenen, K. C., & Gilman, S. E. (2010). Childhood adversity, adult stressful life events, and risk of past-year psychiatric disorder: A test of the stress sensitization hypothesis in a population-based sample of adults. Psychological Medicine, 40(10), 1647–1658. doi:10.1017/S0033291709992121. 20018126 PMC2891275

[ref57] McTeague, L. M., Goodkind, M. S., & Etkin, A. (2016). Transdiagnostic impairment of cognitive control in mental illness. Journal of Psychiatric Research, 83, 37–46. 10.1016/j.jpsychires.2016.08.001. 27552532 PMC5107153

[ref58] Millan, M. J., Agid, Y., Brüne, M., Bullmore, E. T., Carter, C. S., Clayton, N. S., … DeRubeis, R. J. (2012). Cognitive dysfunction in psychiatric disorders: Characteristics, causes and the quest for improved therapy. Nature Reviews Drug Discovery, 11(2), 141. 10.1038/nrd3628. 22293568

[ref59] Mundt, J. C., Marks, I. M., Shear, M. K., & Greist, J. M. (2002). The work and social adjustment scale: A simple measure of impairment in functioning. British Journal of Psychiatry, 180(5), 461–464. 10.1192/bjp.180.5.461. 11983645

[ref60] Nieman, D. H., Chavez-Baldini, U., Vulink, N. C., Smit, D. J. A., van Wingen, G., de Koning, P., … Denys, D. (2020). Protocol across study: Longitudinal transdiagnostic cognitive functioning, psychiatric symptoms, and biological parameters in patients with a psychiatric disorder. BMC Psychiatry, 20(1), 212. doi:10.1186/s12888-020-02624-x. 32393362 PMC7216345

[ref61] Nolen-Hoeksema, S., & Watkins, E. R. (2011). A heuristic for developing transdiagnostic models of psychopathology: Explaining multifinality and divergent trajectories. Perspectives on Psychological Science, 6(6), 589–609. doi:10.1177/1745691611419672. 26168379

[ref62] Ofrat, S., & Krueger, R. F. (2012). How research on the meta-structure of psychopathology aids in understanding biological correlates of mood and anxiety disorders. Biology of Mood & Anxiety Disorders, 2(1), 13. doi:10.1186/2045-5380-2-13. 22898106 PMC3493350

[ref63] Olbert, C. M., Gala, G. J., & Tupler, L. A. (2014). Quantifying heterogeneity attributable to polythetic diagnostic criteria: Theoretical framework and empirical application. Journal of Abnormal Psychology, 123(2), 452. https://psycnet.apa.org/doi/10.1037/a0036068. 24886017 10.1037/a0036068

[ref64] Pechtel, P., & Pizzagalli, D. A. (2011). Effects of early life stress on cognitive and affective function: An integrated review of human literature. Psychopharmacology, 214(1), 55–70. doi:10.1007/s00213-010-2009-2. 20865251 PMC3050094

[ref65] Pons, P., & Latapy, M. (2006). Computing communities in large networks using random walks. Journal of Graph Algorithms and Applications, 10(2), 191–218. 10.1007/11569596_31.

[ref66] Quarantini, L. C., Torres, A. R., Sampaio, A. S., Fossaluza, V., Mathis, M. A. D., do Rosário, M. C., … Koenen, K. C. (2011). Comorbid major depression in obsessive-compulsive disorder patients. Comprehensive Psychiatry, 52(4), 386–393. 10.1016/j.comppsych.2010.09.006. 21087765

[ref67] R Core Team (2020). R: A language and environment for statistical computing. Vienna, Austria: R Foundation for Statistical Computing. Retrieved from https://www.R-project.org.

[ref68] Roiser, J. P., & Sahakian, B. J. (2013). Hot and cold cognition in depression. CNS Spectrums, 18(3), 139–149. 10.1017/S1092852913000072. 23481353

[ref69] Rombouts, R., & Van-Kuilenburg, C. J. (1988). Hedonie, de ontwikkeling van een vragenlijst [Development of a questionnaire designed to measure hedonism]. Gedrag en Gezondheid, 16, 117–123.

[ref70] Rush, A. J., Giles, D. E., Schlesser, M. A., Fulton, C. L., Weissenburger, J., & Burns, C. (1986). The inventory for depressive symptomatology (IDS): Preliminary findings. Psychiatry Research, 18(1), 65–87. 10.1016/0165-1781(86)90060-0. 3737788

[ref71] Sandberg, M.A. (2011) Cambridge neuropsychological testing automated battery. In Kreutzer, J. S., DeLuca, J., & Caplan, B. (Eds.), Encyclopedia of clinical neuropsychology (p. 32). New York, NY: Springer. 10.1007/978-0-387-79948-3_169.

[ref72] Saunders, J. B., Aasland, O. G., Babor, T. F., De la Fuente, J. R., & Grant, M. (1993). Development of the alcohol use disorders identification test (AUDIT): WHO collaborative project on early detection of persons with harmful alcohol consumption-II. Addiction, 88(6), 791–804. 10.1111/j.1360-0443.1993.tb02093.x. 8329970

[ref73] Schmand, B., Bakker, D., Saan, R., & Louman, J. (1991). The Dutch reading test for adults: A measure of premorbid intelligence level. Tijdschrift voor Gerontologie en Geriatrie, 22(1), 15–19. 1877068

[ref74] Scott, J. C., Harb, G., Brownlow, J. A., Greene, J., Gur, R. C., & Ross, R. J. (2017). Verbal memory functioning moderates psychotherapy treatment response for PTSD-related nightmares. Behaviour Research and Therapy, 91, 24–32. 10.1016/j.brat.2017.01.004. 28110112

[ref75] Semkovska, M., Quinlivan, L., O'Grady, T., Johnson, R., Collins, A., O'Connor, J., … Gload, T. (2019). Cognitive function following a major depressive episode: A systematic review and meta-analysis. The Lancet Psychiatry, 6(10), 851–861. 10.1016/S2215-0366(19)30291-3. 31422920

[ref76] Shamsi, S., Lau, A., Lencz, T., Burdick, K. E., DeRosse, P., Brenner, R., … Malhotra, A. K. (2011). Cognitive and symptomatic predictors of functional disability in schizophrenia. Schizophrenia Research, 126(1), 257–264. 10.1016/j.schres.2010.08.007. 20828991 PMC3050077

[ref77] Silverstone, P. H., & Salsali, M. (2003). Low self-esteem and psychiatric patients: Part I – The relationship between low self-esteem and psychiatric diagnosis. Annals of General Hospital Psychiatry, 2(1), 2. doi:10.1186/1475-2832-2-2. 12620127 PMC151271

[ref78] Simon, A. E., Grädel, M., Cattapan-Ludewig, K., Gruber, K., Ballinari, P., Roth, B., & Umbricht, D. (2012). Cognitive functioning in at-risk mental states for psychosis and 2-year clinical outcome. Schizophrenia Research, 142(1), 108–115. 10.1016/j.schres.2012.09.004. 23025995

[ref79] Snyder, H. R., Miyake, A., & Hankin, B. L. (2015). Advancing understanding of executive function impairments and psychopathology: Bridging the gap between clinical and cognitive approaches. Frontiers in Psychology, 6, 328. 10.3389/fpsyg.2015.00328. 25859234 PMC4374537

[ref80] Sowislo, J. F., & Orth, U. (2013). Does low self-esteem predict depression and anxiety? A meta-analysis of longitudinal studies. Psychological Bulletin, 139(1), 213. https://psycnet.apa.org/doi/10.1037/a0028931. 22730921 10.1037/a0028931

[ref81] van Borkulo, C. D., Boschloo, L., Borsboom, D., Penninx, B. W., Waldorp, L. J., & Schoevers, R. A. (2015). Association of symptom network structure with the course of depression. JAMA Psychiatry, 72(12), 1219–1226. 26561400 10.1001/jamapsychiatry.2015.2079

[ref82] van Rooijen, G., Isvoranu, A.M., Kruijt, O. H., van Borkulo, C. D., Meijer, C. J., Wigman, J. T., … Cahn, W. (2017). A state-independent network of depressive, negative and positive symptoms in male patients with schizophrenia spectrum disorders. Schizophrenia Research, 193, 232–239. 10.1016/j.schres.2017.07.035. 28844638

[ref83] Volkow, N. D., Swanson, J. M., Evins, A., DeLisi, L. E., Meier, M. H., Gonzalez, R., … Baler, R. (2016). Effects of cannabis use on human behavior, including cognition, motivation, and psychosis: A review. JAMA Psychiatry, 73(3), 292–297. doi:10.1001/jamapsychiatry.2015.3278. 26842658

[ref84] Weiss, D., & Marmar, C. (1997). The impact of event scale-revised. In J. P. Wilson, & T. M. Keane (Eds.), Assessing psychological trauma and PTSD (pp. 399–411). New York, NY, USA: Guilford Press.

[ref85] Wingenfeld, K., & Wolf, O. T. (2014). Stress, memory, and the hippocampus. In K. Szabo, & M. G. Hennerici (Eds.), The hippocampus in clinical neuroscience (Vol. 34, pp. 109–120). Basel, Switzerland: Karger Publishers. 10.1159/000356423 24777135

[ref86] Zeigler-Hill, V. (2011). The connections between self-esteem and psychopathology. Journal of Contemporary Psychotherapy, 41(3), 157–164. 10.1007/s10879-010-9167-8.

