# Supplementary material for: The relationship between cognitive functioning and psychopathology in patients with psychiatric disorders: a transdiagnostic network analysis
Source: Psychol Med. 2021 Jun 24;53(2):476–85. doi: 10.1017/S0033291721001781 (PMC9899564; doi:10.1017/S0033291721001781)
Supplement: Supplementary file 1 [file S0033291721001781sup001.docx]

Supplementary Materials

Table of Contents

[Appendix 1. Methods 2](#_Toc57197270)

[Stability check 2](#_Toc57197271)

[Control networks for cluster analysis 2](#_Toc57197272)

[Control networks for the interaction between cognition and psychopathology 3](#_Toc57197273)

[Appendix 2. Control network results 3](#_Toc57197274)

[Cluster control conditions 3](#_Toc57197275)

[Control networks for the interaction between cognition and psychopathology 3](#_Toc57197276)

[Table S1. Detailed overview of instruments 4](#_Toc57197277)

[Table S2. Cognitive and psychopathology domain scores of all patients (original scaling): Mean (SD), range 5](#_Toc57197278)

[Table S3. CANTAB Standard Scores 7](#_Toc57197279)

[Table S4. Cognitive domain scores and medication: Mean (SD) 8](#_Toc57197280)

[Table S5. Edge weights of the main network. 10](#_Toc57197281)

[Table S6. Edge weights of the associations between cognitive domains and psychopathology domains^a^ 11](#_Toc57197282)

[Table S7. Results of the bootstrap analysis of the EGA clustering^a^ 12](#_Toc57197283)

[Table S8. Results of the bootstrap analysis of the EGA clustering- Node placement^a^ 12](#_Toc57197284)

[Figure S1. Strength centrality measure of psychopathology and cognitive domains. 13](#_Toc57197285)

[Figure S2. Accuracy of the estimated edges of the network. 14](#_Toc57197286)

[Figure S3. Difference tests of edges in the network 15](#_Toc57197287)

[Figure S4. Difference test of total strength per node in the network 16](#_Toc57197288)

[Figure S5. Stability of centrality indices of the network after ‘dropping’ a part of the patients 17](#_Toc57197289)

[Figure S6. Networks for substance use fused and no zero substance use 18](#_Toc57197290)

[Figure S7. Accuracy of the estimated edges of substance use control networks 18](#_Toc57197291)

[Figure S8. Networks of subsamples by demographics: education level, sex, and age 19](#_Toc57197292)

[Figure S9. Accuracy of the estimated edges of demographic control networks 20](#_Toc57197293)

[Figure S10. Networks of diagnostic subsamples 21](#_Toc57197294)

[Figure S11. Accuracy of the edges of diagnostic subsample control networks 22](#_Toc57197295)

[Figure S12. Networks of medication use subsamples 23](#_Toc57197296)

[Figure S13. Accuracy of the edges of medication use subsample control networks 23](#_Toc57197297)

[Figure S14. Ising network of 690 patients using binarized data and the IsingFit package 24](#_Toc57197298)

[References 25](#_Toc57197299)

# **Appendix 1.** Methods

## **Stability check**

We performed a stability check implemented in the R package ‘bootnet’ to assess the robustness of the networks we estimated; the full procedure has been described previously with instructions of Epskamp, Borsboom, and Fried (2018). Altogether, this stability check allows one to assess three different aspects of the estimated network: (1) the accuracy of edge weights, (2) test for differences between different edges and different centralities and (3) assess the stability of centralities.

The procedure of (1) and (2) involve a method called non-parametric bootstrapping (hereafter simply referred to as bootstrapping). Bootstrapping is a method in which one generates a new sample by sampling from one’s data with replacement (Efron & Tibshirani, 1994). This means that you can draw a certain patient multiple times and that the probability of sampling a patient does not change after sampling that patient. For example, if your data consists out of 100 patients {p_1_, p_2_… p_100_} , the probability of selecting any patient p_x_ after you start and selected the first patient remains 1/100, whereas whilst sampling without replacement it would become 0 for the patient p_x_ already selected, and 1/99 for all the remaining patients {p_1_... p_x-1_, p_x+1_… p_100_}. This procedure results in a new sample that has the same size as the original sample but contains certain patients multiple times. This procedure can be repeated n times, to generate n new samples, in our case we used n = 1000 to generate 1000 new samples.

The next step is to estimate a network for each of the new samples using the same EBICglasso procedure (Chen & Chen, 2008; Foygel & Drton, 2010; Friedman, Hastie, & Tibshirani, 2008). These 1000 networks can be used to generate a distribution of various network attributes, such as edge weights. A 95% confidence interval of the edge weights can be obtained by sorting the 1000 values from the networks and taking the 0.025^th^ and the 0.975^th^ value. The width of this interval is an indication of (1) the accuracy of the edge weights (see Figure S2 below).

To obtain (2) the difference tests for edge weights (for example comparing edge A-B to edge A-C) one uses the same 1000 networks obtained from bootstrapping, but this time one calculates the differences between two edges in each network. These 1000 differences can be used to construct a 95% confidence interval in the same way as described above. If the interval does not contain zero this means that the edges differ at the α = 0.05 level (Figure S2 below contains a visualization of the results of this test). The same procedure can be used to test differences between the strength of two nodes (see Figure S3 below for a visualization).

The final procedure to (3) assess the stability of the centralities of the network is a bit different from above. This procedure uses a method called the *m out of n bootstrap* (Chernick, 2007). In this procedure, a certain percentage of the sample is randomly removed, ‘dropped’, to yield a new sample, which can be repeated a certain number of times. The next step is the estimation of a network for each sample and the calculation of centralities (Opsahl, Agneessens, & Skvoretz, 2010). The metric of interest that will be calculated next is the correlation between the centralities of each ‘dropped’ network and the centralities of the original network, this correlation is an indication of the stability of the order of the centralities rather than a measure of the absolute sameness of the centralities (Epskamp et al., 2018). Correlations above 0.7 are acceptable and indicate that the centralities are still stable after dropping the given percentage of people.

This means that the centralities are considered stable up to a point where dropping that percentage of patients will include a correlation of 0.7 in the 95% confidence interval. Figure S5 is a visualization of the average correlation to the original centralities after dropping a certain percentage of patients and the shaded areas indicate the 95% confidence intervals.

We also followed a similar procedure to assess the stability of the results of the clustering analysis using EGA. The *EGA* package has the function *bootEGA* which uses non-parametric bootstrapping based on resampling, the same as used in procedures (1) and (2) above (Golino & Epskamp, 2017). By resampling and performing the EGA analysis 1000 times allows one to get a distribution of the number of dimensions in the bootstraps of a network. We performed this procedure for the main network (Figure 1 in the main article) and for the networks that were created as controls for the cluster analysis (see paragraph 1.2 below for further explanation of these networks). The results of the cluster stability analysis can be seen in Table S7.

## **Control networks for cluster analysis**

The cluster analysis of the main network (Figure 1 in the main article) revealed one minor cluster: substance use. There are two factor that may have influenced the formation of these clusters: (a) many of the patients in our sample had no scores (i.e., zero’s) on any of the domains in this cluster and (b) the subdomains of this cluster may have been separated unfairly and should have left together in one variable since the subdomains essentially measure the same construct. To control for these possible confounding situations, we created two control networks for each this cluster: one in which all patients were removed that have only zero’s in all the items in that cluster and another one where the subdomains were not separated. We conducted all the analyses for the control networks in the same way as was done for the unchanged network: network estimation, stability analysis, cluster detection, and cluster stability analysis as described in the supplementary method above. The resulting networks can be seen in Figure S6, the stability analysis of the edges can be seen in Figure S7 and the results of the cluster stability analysis can be seen in Table S7.

## **Control networks for the interaction between cognition and psychopathology**

The main analysis exhibited a pattern in which cognition variables interacted mostly with other cognition variables and psychopathology variables interacted mostly with other psychopathology variables. There were two possible confounding properties of the data that could artificially generate such a pattern: (a) differences in measurement methods and used scales between the cognitive and psychopathological measures could result in different distributions in the variables and may thereby result in weaker network edges between those domains, and (b) sample variation in the level of cognitive functioning could weaken the statistical covariation of cognition with psychopathology.

In order to control for the first possibility (a), we binarized the data by setting for each variable all values that were above the median of that variable to 1 and everything equal or below the median to zero. Binarization thereby removes any differences between cognitive and psychopathological variables in distribution or scaling. Networks were computed from the binarized data using the *IsingFit* function from the *IsingFit* package, was explicitly developed as a binary alternative to the partial correlation method (Van Borkulo et al., 2014). The resulting network can be seen in Figure S12.

In order to control for the second possibility (b) we split the data into two groups based on prior educational level: high and low (Verhage, 1964). This resulted in two groups with a more similar level of prior cognitive functioning and might reveal more covariation between cognitive functioning and psychopathology. We computed networks for these groups using the same procedure as the main analysis. Similar analyses were conducted for male versus female patients and younger (up to 31 years old) versus older (31 years old and above) patients. Finally, to assess the impact of patient diagnosis on cluster structure we conducted a jackknife sensitivity analysis by leaving out patients belonging to 6 major categories: anxiety disorders, depressive disorders, misophonia, obsessive-compulsive and related disorders, schizophrenia spectrum and other psychotic disorders, and other disorders.

# **Appendix 2.** Control network results

## **Cluster control conditions**

In our main analysis, we detected 1 smaller cluster: substance use. We noted that this cluster may have been formed due to confounding factors: many patients with only zeros on the substance use items or the undue splitting of substance use into cannabis use and alcohol. To control for these potential confounding factors, we created two control conditions for the substance use cluster: removing patients with zeros on all items in the cluster and conducting the analysis without splitting substance use into subscales. The results of the control conditions can be seen in Figure S6. To assess the stability of the clustering in these networks, we furthermore conducted a bootstrapping analysis as described above, the results of which can be seen in Table S7. Fusing of the cannabis and alcohol use scales resulted in fusion of the substance use cluster with the psychopathology cluster. Interestingly, removing the patients that had zeros on both substance use scales resulted in the same cluster structure as in the original network.

The analysis of these control conditions shows how important it is to be careful with choices regarding the inclusion and fusion of nodes in the network. Undue fusion of two nodes that are in fact too dissimilar will result in loss of signal due to the mixing, while undue separation of subscales will create a very strong separate cluster. Therefore, it is important to incorporate cluster stability analysis, and to plot and check all the different networks to assess their feasibility.

## **Control networks for the interaction between cognition and psychopathology**

The Ising network (Figure S12) estimated from binarized data to control for confounding effects of differences in variable distribution revealed a very weak pattern of interaction between cognition and psychopathology. This leads us to conclude that differences in variable distribution between cognitive and psychopathological variables did not reduce the pattern of interaction that we found.

The EBICglasso networks that we estimated for patients with a higher educational level (Figure S8a) and with a lower educational level (Figure S8b) separately did not reveal differences in cluster structure regarding cognitive and psychopathological variables. This leads us to conclude that prior differences in educational level did not reduce the pattern of interaction that we found between cognition and psychopathology. Similarly, control networks for male (Figure S8c) versus female (Figure S8d) patients and younger (up to 31, Figure S8e) versus older (31 and above, Figure S8f) patients demonstrated no differences in cluster structure. Finally, diagnostic categories also did not reveal differences in cluster structure regarding cognitive and psychopathological variables (Figure S10): anxiety disorders (10a), depressive disorders (10b), misophonia (10c), obsessive-compulsive and related disorders (10d), schizophrenia spectrum and other psychotic disorders (10e), and other disorders (10f).

# **Table S1.** Detailed overview of instruments

| **Instrument** | | **Description** | **Outcome Measure** |
| --- | --- | --- | --- |
| Cambridge Neuropsychological Test Automated Battery (CANTAB; Cambridge Cognition, 2018) | | Assesses cognitive functioning and is composed of the following subtests: |  |
|  | Verbal Recognition Memory-immediate (VRM) | Assesses immediate free recall and recognition memory for verbal information | Free recall- total correct:  Total number of distinct words correctly recalled. |
|  | Rapid Visual Information Processing (RVP) | Tests visual sustained attention | A′:  Measure of how good the subject is at detecting target sequences. |
|  | Intra/ Extradimensional Set Shift (IED) | Assesses rule acquisition and attentional set shifting | Completed stage errors:  Number of intra-dimensional and extra-dimensional shift errors made on stages successfully completed. |
|  | Choice reaction time (CRT) | Measures alertness and motor speed | Mean correct latency:  Mean latency of response (from stimulus appearance to button press). |
|  | Verbal Recognition Memory (VRM-delayed) | Assesses delayed recognition memory for verbal information | Recognition- total correct:  Total number of words that the subject correctly recognizes. |
|  | One Touch Stockings of Cambridge (OTS) | A planning test which gives a measure of frontal lobe functioning | Problems solved on first choice:  Number of problems which were solved on the subject’s first  choice. |
|  | Paired Associates Learning (PAL) | Assesses episodic memory and learning | Total errors (adjusted):  Total number of errors adjusted for number of trials completed. |
|  | Spatial Working Memory (SWM) | Assesses working memory and strategy use | Strategy:  Number of times the subject begins a new search. |
| Dutch National Adult Reading Test (NART; Schmand, Bakker, Saan, & Louman, 1991) | | Measures premorbid IQ | Total score |
| California Verbal Learning Test (CVLT; Delis, Kramer, Kaplan, & Ober, 2000) | | Assesses episodic verbal learning and memory | Performance level List A:  Overall performance level on the first 5 trials. |
| Groninger Intelligence Test (GIT; Luteijn & Van der Ploeg, 1983) | | Measures level of verbal fluency | Number of words named (animal category) |
| Alcohol Use Disorder Identification Test (AUDIT; Saunders, Aasland, Babor, De la Fuente, & Grant, 1993) | | Assesses alcohol consumption, drinking behaviors, and alcohol-related problems | Total score |
| Cannabis Use Disorder Identification Test (CUDIT; Adamson & Sellman, 2003) | | Screens for cannabis use problems | Total score |
| Prodromal Questionnaire-16 (PQ-16; Ising et al., 2012) | | Screens for the risk of psychosis | Total score |
| Yale-Brown Obsessive Compulsive Scale (Y-BOCS; Goodman et al., 1989) | | Measures the severity and type of obsessive-compulsive symptoms | Total score |
| Hamilton Anxiety Scale (HAM-A; Hamilton, Schutte, & Malouff, 1976) | | Assess severity of somatic, cognitive, and affective symptoms in anxiety | Total score |
| Inventory of Depressive Symptomology Self-Report (IDS-SR; Rush et al., 1986) | | Measures the severity of depressive symptoms | Total score |
| Impact of Events Scale- Revised (IES-R; Weiss, Marmar, Wilson, & Keane, 1997) | | Assesses subjective distress caused by traumatic events | Total score |
| Hedonism Scale (Rombouts & Van-Kuilenburg, 1988) | | Measures degree of pleasure from physical activity, hearing, seeing, touching, tasting, sex and smelling | Total score |
| Self-esteem Rating Scale- Short Form (SERS-SF; Lecomte, Corbière, & Laisné, 2006) | | Assesses self-esteem in relation to social contact, achievements, and competency | Total score |
| Work and Social Adjustment Scale (WSAS; Mundt, Marks, Shear, & Greist, 2002) | | Measures general impairment in different domains of daily life | Total score |
| Social Interaction Anxiety Scale (SIAS; Mattick & Clarke, 1998) | | Assess anxiety in social interactions and fear of scrutiny by others | Total score |

# **Table S2.** Cognitive and psychopathology domain scores of all patients (original scaling): Mean (SD), range

| **Measure** | **All patients (N=1016)** | **SZ (n=185)** | **Depressive disorders (n=111)** | **Anxiety disorders (n=60)** | **OCD (n=177)** | **Misophonia (n=353)** | **Other disorders^a^ (n=130)** |
| --- | --- | --- | --- | --- | --- | --- | --- |
| Anxiety symptoms (HAM-A)^b^ | 13.24 (10.0),  0-52 | 9.27 (9.2), 0-52 | 19.42 (9.8), 1-50 | 21.09 (10.6), 1-42 | 16.73 (10.0), 0-45 | 9.97 (7.8), 0-36 | 13.88 (10.4), 0-40 |
| Depressive symptoms (IDS-SR) ^b^ | 23.13 (14.0),  0-72 | 19.37 (13.2), 0-59 | 36.63 (14.2), 0-72 | 29.12 (13.0), 4-57 | 25.23 (12.4), 3-68 | 17.35 (11.0), 0-53 | 24.90 (13.6), 1-59 |
| Subclinical psychotic symptoms (PQ-16)^b^ | 5.56 (5.7), 0-33 | 7.03 (7.3), 0-33 | 6.95 (6.5), 0-29 | 6.75 (5.5), 0-24 | 5.50 (6.0), 0-31 | 4.18 (4.1), 0-22 | 5.77 (5.7), 0-32 |
| Alcohol use (AUDIT)^b^ | 5.15 (4.9), 0-37 | 5.40 (6.0), 0-37 | 4.28 (4.1), 0-17 | 3.88 (4.2), 0-19 | 4.22 (4.3), 0-24 | 5.56 (4.0), 0-22 | 6.42 (6.6), 0-35 |
| Cannabis use (CUDIT)^b^ | 1.51 (4.7), 0-35 | 4.46 (8.1), 0-33 | 1.10 (2.9), 0-19 | 0.80 (3.4), 0-24 | 0.64 (2.1), 0-14 | 0.43 (1.8), 0-16 | 2.33 (6.2), 0-35 |
| Self-esteem (SERS-SF)^c^ | 12.19 (20.2),  -54- 60 | 15.84 (20.0),  -48- 58 | -1.00 (18.2), -45-45 | 9.71 (22.6), -44- 50 | 6.73 (20.7), -54 - 45 | 18.88 (17.6), -46 - 60 | 9.55 (18.9), -32 - 53 |
| Social anxiety (SIAS)^b^ | 23.55 (15.4),  0-75 | 20.00 (13.9), 0-66 | 32.96 (15.7), 2-69 | 26.88 (18.7), 3-66 | 26.40 (15.9), 0-75 | 19.79 (13.0), 0-62 | 24.94 (15.6), 0-60 |
| Psychosocial functioning (WSAS)^b^ | 17.43 (8.9), 0-40 | 17.61 (9.1), 0-40 | 20.94 (9.4), 2-39 | 17.85 (8.2), 0-36 | 19.46 (9.2), 2-40 | 15.06 (7.9), 0-34 | 17.55 (9.0), 0-36 |
| Hedonism Scale^c^ | 58.07 (11.2),  21-84 | 59.35 (11.3), 21-84 | 49.07 (10.6), 29-78 | 53.14 (12.7), 21-80 | 57.31 (10.4), 25-84 | 62.02 (9.6), 21-83 | 57.07 (10.0), 23-83 |
| OC symptoms (Y-BOCS)^b^ | 6.61 (9.5), 0-39 | 3.37 (7.4), 0-34 | 5.21 (8.1), 0-31 | 8.48 (9.9), 0-37 | 17.25 (10.4), 0-38 | 3.23 (6.4), 0-39 | 6.07 (8.2), 0-32 |
| PTS symptoms (IES-R)^b^ | 9.40 (17.7), 0-88 | 10.42 (18.4), 0-81 | 17.05 (22.4), 0-72 | 16.64 (22.6), 0-68 | 8.63 (17.3), 0-88 | 5.69 (13.5), 0-69 | 9.27 (17.2), 0-65 |
| CRT Mean correct latency^b^ | 343.61 (93.0), 220-1629 | 345.21 (99.2), 229-1168 | 372.82 (100.3), 233–806 | 356.65 (118.4), 220–971 | 346.85 (86.9), 221–953 | 328.56 (59.5), 223–716 | 346.90 (133.5), 225-1629 |
| RVP A’^c^ | 0.90 (0.1),  0.6- 1.0 | 0.87 (0.1), 0.6-1.0 | 0.89 (0.1), 0.7–1.0 | 0.89 (0.1), 0.8– 1.0 | 0.90 (0.1), 0.8–1.0 | 0.91 (0.1), 0.8–1.0 | 0.90 (0.1), 0.7-1.0 |

**Table S2.** Cognitive and psychopathology domain scores of all patients (original scaling): Mean (SD), range (continued)

| **Measure** | **All patients (N=1016)** | **SZ (n=185)** | **Depressive disorders (n=111)** | **Anxiety disorders (n=60)** | **OCD (n=177)** | **Misophonia (n=353)** | **Other disorders^a^ (n=130)** |
| --- | --- | --- | --- | --- | --- | --- | --- |
| VRM free recall-immediate^c^ | 6.95 (2.7), 0-16 | 6.45 (2.3), 0-15 | 6.39 (2.8), 1–16 | 6.88 (2.8), 3–14 | 6.90 (3.0), 0–14 | 7.43 (2.5), 0–15 | 6.94 (2.7), 0-14 |
| VRM recognition-delayed^c^ | 32.36 (3.0), 20-36 | 32.30 (3.0), 20-36 | 31.46 (3.4), 21–36 | 32.05 (3.1), 21–36 | 32.47 (3.0), 20–36 | 32.76 (2.6), 24–36 | 32.21 (3.2), 20-36 |
| Premorbid IQ (NART)^c^ | 100.09 (13.3), 36-143 | 93.28 (13.8), 36-127 | 101.25 (14.5), 50–131 | 102.05 (12.2), 80–135 | 102.21 (14.5), 55–143 | 101.70 (11.1), 66–129 | 100.47 (12.9), 63-127 |
| PAL Total errors adjusted^b^ | 13.21 (20.1), 0-129 | 18.59 (25.9), 0-129 | 19.13 (25.1), 0–118 | 15.45 (23.9), 0–126 | 14.73 (21.1), 0–116 | 8.11 (12.0), 0–115 | 11.21 (16.3), 0-125 |
| OTS Problems solved on first choice^c^ | 17.61 (3.3), 2-24 | 16.56 (4.1), 3-24 | 17.5 (3.3), 9–23 | 17.68 (3.1), 10–23 | 17.01 (3.2), 6–23 | 18.51 (2.7), 9–23 | 17.45 (3.5), 2-23 |
| SWM Strategy use^b^ | 31.31 (6.3), 18-48 | 32.66 (6.2), 18-44 | 31.39 (6.9), 19–44 | 31.05 (6.1), 19–43 | 32.77 (6.1), 19–46 | 29.85 (6.10), 18–48 | 31.45 (6.4), 18-48 |
| IED Completed stage errors^b^ | 13.15 (8.0), 0-59 | 15.26 (9.2), 0-54 | 12.05 (6.5), 2–42 | 12.80 (8.6), 2–59 | 12.86 (8.3), 1–53 | 12.90 (7.3), 2–42 | 12.32 (7.6), 1-44 |
| Verbal memory (CVLT)^c^ | 57.66 (11.3), 18-80 | 50.10 (11.8), 18-75 | 57.34 (11.0), 25–79 | 58.67 (10.6), 30–80 | 60.01 (9.9), 24–79 | 61.01 (9.2), 30–76 | 56.16 (12.5), 20-78 |
| Verbal fluency (GIT)^c^ | 24.22 (6.4), 7-49 | 19.92 (6.2), 7-41 | 23.81 (5.7), 10–42 | 23.54 (6.4), 11–39 | 26.07 (6.7), 9–41 | 25.78 (5.7), 13–49 | 24.13 (6.9), 7-41 |

Abbreviation: SZ= Schizophrenia spectrum and other psychotic disorders, OCD= Obsessive-compulsive and related disorders, Misophonia= Impulse-control disorder NOS (misophonia), AUDIT= Alcohol Use Disorder Identification Test, CUDIT = Cannabis Use Disorder Identification Test, PQ-16 = Prodromal Questionnaire, Y-BOCS = Yale-Brown Obsessive Compulsive Scale, HAM-A = Hamilton Anxiety Scale, IDS-SR = Inventory of Depressive Symptomology Self-Report, IES-R = Impact of Events Scale- Revised, SERS-SF = Self-esteem Rating Scale- Short Form, WSAS = Work and Social Adjustment Scale, SIAS = Social Interaction Anxiety Scale, CRT = Choice Reaction Time, CLVT= California Verbal Learning Test, GIT= Groninger Intelligence Test, IED = Intra/ Extradimensional Set Shift, NART= Dutch National Adult Reading Test, OTS = One Touch Stockings of Cambridge, PAL = Paired Associates Learning, RVP = Rapid Visual Processing, SWM = Spatial Working Memory, VRM = Verbal Recognition Memory.

^a^ Other disorder category includes: substance use disorders, eating disorders, neurodevelopmental disorders, sexual disorders, sleep disorders, dissociative disorders, adjustment disorders, bipolar disorders, and personality disorders.
^b^ Scaling: higher is worse.
^c^ Scaling: higher is better.

# **Table S3.** CANTAB Standard Scores^a^

| **Variable** | **Standard Score (M, SD)** | **Range** | **Deficit^b^, yes, No. (%)** |
| --- | --- | --- | --- |
| CRT Mean correct latency^c^ | - | - | - |
| RVP A’ | -0.42 (1.2) | -5.95 – 2.50 | 202 (19.9) |
| VRM free recall-immediate^c^ | - | - | - |
| VRM recognition-delayed^c^ | - | - | - |
| PAL Total errors adjusted | -0.0023 (1.8) | -23.03 – 1.52 | 83 (8.2) |
| OTS Problems solved on first choice^c^ | - | - | - |
| SWM Strategy use | 0.16 (1.1) | -2.94 – 3.73 | 61 (6.0) |
| IED Completed stage errors | -0.03 (1.1) | -7.56 – 2.08 | 116 (11.4) |

Abbreviation: CRT = Choice Reaction Time, IED = Intra/ Extradimensional Set Shift, OTS = One Touch Stockings of Cambridge, PAL = Paired Associates Learning, RVP = Rapid Visual Processing, SWM = Spatial Working Memory, VRM = Verbal Recognition Memory.

^a^ Standard scores should be interpreted with caution as they are calculated with normative data that is not matched by age and premorbid IQ>

^b^Deficit: no > -1.5, yes < -1.5

^c^No normative data available.

# **Table S4**. Cognitive domain scores and medication (original scaling): Mean (SD)

| **Variable** | **None (n= 392)** | **Anti-depressants (n= 213)** | **Benzo-diazepines (n= 30)** | **Anti-psychotics (n= 169)** | **Mood stabilizers (n= 8)** | **Psycho-stimulants (n= 8)** | **Sleep medication (n= 15)** | **Other^a^ (n= 168)** | **Test Statistic** | **p** |
| --- | --- | --- | --- | --- | --- | --- | --- | --- | --- | --- |
| Anxiety symptoms (HAM-A)^b^ | 11.55 (9.3) | 16.42 (9.4) | 20.84 (10.8) | 10.15 (9.7) | 15.29 (8.1) | 20.86 (12.4) | 20.93 (10.1) | 13.36 (10.4) | F= 11.29, df= 7 | **<.001** |
| Depressive symptoms (IDS-SR)^b^ | 20.02 (12.7) | 28.74 (13.7) | 30.11 (14.8) | 20.92 (13.6) | 31.14 (13.9) | 33.00 (21.8) | 34.07 (15.1) | 22.65 (14.0) | F= 11.84, df= 7 | **<.001** |
| Subclinical psychotic symptoms (PQ-16)^b^ | 3.50 (2.9) | 3.84 (3.2) | 4.70 (2.9) | 4.61 (3.7) | 3.29 (2.1) | 6.14 (5.2) | 4.85 (3.3) | 3.61 (3.1) | F= 3.00, df= 7 | **.004** |
| Alcohol use (AUDIT)^b^ | 5.51 (4.9) | 4.63 (4.9) | 4.40 (4.5) | 5.60 (6.0) | 4.29 (5.7) | 5.43 (3.3) | 4.79 (3.7) | 4.71 (3.7) | F= 1.10, df= 7 | .359 |
| Cannabis use (CUDIT)^b^ | 1.30 (4.6) | 1.07 (3.6) | 2.20 (4.6) | 3.56 (7.3) | 0.57 (1.5) | 1.00 (2.6) | 1.07 (2.8) | 0.45 (1.7) | F= 6.03, df= 7 | **<.001** |
| Self-esteem (SERS-SF)^c^ | 16.30 (19.3) | 6.66 (20.3) | 11.44 (19.2) | 12.40 (21.2) | 5.00 (18.5) | 5.14 (16.6) | 3.86 ( 21.0) | 11.07 (19.7) | F= 5.27, df= 7 | **<.001** |
| Social anxiety (SIAS)^b^ | 21.26 (14.4) | 27.40 (16.3) | 24.48 (15.0) | 21.92 (15.2) | 28.43 (15.6) | 32.00 (19.1) | 30.93 (19.1) | 24.41 (15.2) | F= 4.34, df= 7 | **<.001** |
| Psychosocial functioning (WSAS)^b^ | 16.15 (8.6) | 18.92 (8.7) | 18.62 (9.0) | 17.74 (9.5) | 19.29 (7.0) | 15.43 (10.2) | 22.15 (8.8) | 17.22 (8.6) | F= 2.65, df= 7 | **.01** |
| Hedonism Scale^c^ | 59.88 (10.9) | 54.31 (11.1) | 55.27 (11.6) | 58.55 (12.1) | 48.14 (9.1) | 54.14 (10.3) | 54.64 (12.9) | 59.66 (9.3) | F= 6.82, df= 7 | **<.001** |
| OC symptoms (Y-BOCS)^b^ | 5.25 (8.3) | 10.28 (10.7) | 6.27 (10.6) | 4.94 (9.3) | 8.86 (11.9) | 13.29 (8.7) | 7.93 (9.6) | 6.06 (8.8) | F= 7.23, df= 7 | **<.001** |
| PTS symptoms (IES-R)^b^ | 7.77 (16.0) | 12.00 (19.6) | 21.67 (23.2) | 9.55 (17.4) | 9.86 (26.1) | 10.71 (27.0) | 15.57 (23.8) | 7.22 (15.5) | F= 3.60, df= 7 | **.001** |
| CRT Mean correct latency^b^ | 334.49 (95.1) | 350.98 (99.9) | 378.81 (79.6) | 341.93 (75.6) | 339.68 (37.0) | 371.44 (175.9) | 341.87 (78.5) | 349.97 (95.6) | F= 1.56, df= 7 | .144 |

**Table S4**. Cognitive domain scores and medication (original scaling): Mean (SD), continued

| **Variable** | **None (n= 392)** | **Anti-depressants (n= 213)** | **Benzo-diazepines (n= 30)** | **Anti-psychotics (n= 169)** | **Mood stabilizers (n= 8)** | **Psycho-stimulants (n= 8)** | **Sleep medication (n= 15)** | **Other^a^ (n= 168)** | **Test Statistic** | **p** |
| --- | --- | --- | --- | --- | --- | --- | --- | --- | --- | --- |
| RVP A’ ^c^ | 0.91 (0.1) | 0.89 (0.1) | 0.88 (0.1) | 0.88 (0.1) | 0.86 (0.1) | 0.88 (0.1) | 0.90 (0.1) | 0.90 (0.1) | F= 5.66, df= 7 | **<.001** |
| VRM free recall-immediate^c^ | 7.37 (2.6) | 6.73 (2.8) | 6.23 (2.3) | 6.30 (2.3) | 6.13 (3.5) | 7.63 (3.7) | 6.13 (2.3) | 7.14 (2.8) | F= 3.93, df= 7 | **<.001** |
| VRM recognition-delayed^c^ | 32.65 (2.9) | 31.79 (3.3) | 31.59 (2.5) | 32.10 (2.8) | 33.29 (2.1) | 32.50 (4.7) | 31.43 (2.7) | 32.80 (2.7) | F= 2.97, df= 7 | **.004** |
| Premorbid IQ (NART)^c^ | 100.87 (12.4) | 100.98 (13.4) | 100.80 (14.1) | 95.26 (14.8) | 93.63 (18.0) | 93.38 (7.3) | 95.87 (12.0) | 102.26 (12.4) | F= 4.95, df= 7 | **<.001** |
| PAL Total errors adjusted^b^ | 9.76 (17.5) | 16.97 (23.0) | 20.57 (23.4) | 18.93 (24.1) | 9.13 (7.4) | 5.88 (3.8) | 10.27 (7.3) | 10.55 (16.4) | F= 6.06, df= 7 | **<.001** |
| OTS Problems solved on first choice^c^ | 18.35 (3.0) | 17.25 (3.0) | 15.293 (4.7) | 16.11 (3.9) | 18.33 (2.3) | 19.40 (2.2) | 18.00 (2.4) | 18.08 (2.8) | F= 9.88, df= 7 | **<.001** |
| SWM Strategy use^b^ | 30.12 (6.0) | 31.50 (6.5) | 34.93 (5.3) | 33.12 (6.0) | 35.00 (5.0) | 30.29 (7.0) | 31.53 (5.0) | 31.45 (6.6) | F= 6.08, df= 7 | **<.001** |
| IED Completed stage errors^b^ | 13.29 (8.1) | 12.61 (7.6) | 12.00 (7.9) | 14.04 (8.7) | 14.63 (6.1) | 13.25 (6.6) | 12.53 (6.7) | 13.02 (7.8) | F= 0.60, df= 7 | .757 |
| Verbal memory (CVLT)^c^ | 59.82 (10.1) | 58.97 (10.6) | 55.57 (10.7) | 49.39 (11.9) | 59.00 (10.2) | 60.86 (9.5) | 58.33 (8.0) | 59.60 (10.6) | F= 17.90. df= 7 | **<.001** |
| Verbal fluency (GIT)^c^ | 24.55 (6.1) | 25.26 (6.4) | 22.97 (6.3) | 20.95 (6.5) | 23.71 (6.0) | 23.71 (3.4) | 22.71 (5.0) | 25.77 (6.1) | F= 9.01. df= 7 | **<.001** |

Abbreviation: AUDIT= Alcohol Use Disorder Identification Test, CUDIT = Cannabis Use Disorder Identification Test, HAM-A = Hamilton Anxiety Scale, IDS-SR = Inventory of Depressive Symptomology Self-Report, IES-R = Impact of Events Scale- Revised, PQ-16 = Prodromal Questionnaire, SERS-SF = Self-esteem Rating Scale- Short Form, SIAS = Social Interaction Anxiety Scale, WSAS = Work and Social Adjustment Scale, Y-BOCS = Yale-Brown Obsessive Compulsive Scale; CRT = Choice Reaction Time, CLVT= California Verbal Learning Test, GIT= Groninger Intelligence Test, IED = Intra/ Extradimensional Set Shift, OTS = One Touch Stockings of Cambridge, PAL = Paired Associates Learning, RVP = Rapid Visual Processing, SWM = Spatial Working Memory, VRM = Verbal Recognition Memory.
**^a^** Other medication: Anti-epileptic drugs, anti-inflammatory drugs, antihistamines, bronchodilators, cholesterol medication, contraceptives, corticosteroids, dopamine-agonists, hormone therapy, insulin, and migraine medications.
^b^ Scaling: higher is worse.
^c^ Scaling: higher is better.

# **Table S5.** Edge weights of the main network.

|  | Anx | Depr | Psy | Alc | Can | Self | SoAn | SoFu | Hedo | ObCo | Trau | MoS | ViA | FreR | PIQ | EpM | FrFu | StrU | Dela | RuAc | VeM | VeF |
| --- | --- | --- | --- | --- | --- | --- | --- | --- | --- | --- | --- | --- | --- | --- | --- | --- | --- | --- | --- | --- | --- | --- |
| Anx | 0 |  |  |  |  |  |  |  |  |  |  |  |  |  |  |  |  |  |  |  |  |  |
| Depr | .528 | 0 |  |  |  |  |  |  |  |  |  |  |  |  |  |  |  |  |  |  |  |  |
| Psy | .104 | .201 | 0 |  |  |  |  |  |  |  |  |  |  |  |  |  |  |  |  |  |  |  |
| Alc | 0 | 0 | 0 | 0 |  |  |  |  |  |  |  |  |  |  |  |  |  |  |  |  |  |  |
| Can | 0 | 0 | .042 | .198 | 0 |  |  |  |  |  |  |  |  |  |  |  |  |  |  |  |  |  |
| Self | 0 | .224 | 0 | 0 | 0 | 0 |  |  |  |  |  |  |  |  |  |  |  |  |  |  |  |  |
| SoAn | .028 | .132 | .118 | 0 | 0 | .545 | 0 |  |  |  |  |  |  |  |  |  |  |  |  |  |  |  |
| SoFu | .065 | .149 | 0 | 0 | 0 | 0 | .032 | 0 |  |  |  |  |  |  |  |  |  |  |  |  |  |  |
| Hedo | 0 | .161 | 0 | -.087 | 0 | .11 | .014 | 0 | 0 |  |  |  |  |  |  |  |  |  |  |  |  |  |
| ObCo | .211 | 0 | .083 | -.015 | 0 | 0 | .008 | .045 | 0 | 0 |  |  |  |  |  |  |  |  |  |  |  |  |
| Trau | .045 | .13 | .092 | 0 | 0 | 0 | 0 | 0 | 0 | 0 | 0 |  |  |  |  |  |  |  |  |  |  |  |
| MoS | -.008 | -.009 | -.031 | .021 | 0 | 0 | 0 | 0 | -.056 | 0 | 0 | 0 |  |  |  |  |  |  |  |  |  |  |
| ViA | 0 | 0 | -.033 | .023 | -.028 | 0 | 0 | 0 | 0 | 0 | -.045 | .065 | 0 |  |  |  |  |  |  |  |  |  |
| FreR | 0 | -.011 | -.001 | 0 | 0 | 0 | 0 | 0 | 0 | 0 | 0 | .004 | .054 | 0 |  |  |  |  |  |  |  |  |
| PIQ | 0 | 0 | -.05 | 0 | -.001 | 0 | 0 | 0 | 0 | 0 | -.002 | 0 | .149 | 0 | 0 |  |  |  |  |  |  |  |
| EpM | 0 | 0 | 0 | 0 | 0 | 0 | 0 | 0 | 0 | 0 | 0 | .122 | .055 | .043 | 0 | 0 |  |  |  |  |  |  |
| FrFu | 0 | 0 | 0 | 0 | 0 | 0 | 0 | 0 | 0 | -.006 | 0 | 0 | .131 | 0 | 0 | .145 | 0 |  |  |  |  |  |
| StrU | 0 | 0 | 0 | .05 | 0 | 0 | 0 | 0 | 0 | -.026 | -.017 | .073 | .153 | 0 | 0 | .102 | .202 | 0 |  |  |  |  |
| Dela | 0 | -.037 | -.04 | 0 | 0 | 0 | 0 | 0 | 0 | 0 | 0 | .106 | 0 | .314 | 0 | .104 | 0 | 0 | 0 |  |  |  |
| RuAc | 0 | 0 | 0 | 0 | 0 | 0 | 0 | 0 | 0 | 0 | 0 | 0 | .042 | 0 | 0 | .096 | 0 | 0 | 0 | 0 |  |  |
| VeM | 0 | 0 | -.005 | 0 | -.028 | 0 | .014 | 0 | -.035 | .02 | 0 | .026 | .105 | .219 | .089 | .192 | .119 | .016 | .108 | 0 | 0 |  |
| VeF | 0 | 0 | 0 | .037 | -.037 | 0 | 0 | 0 | -.013 | .024 | 0 | 0 | .134 | .068 | .173 | 0 | .044 | 0 | 0 | .012 | .228 | 0 |

Abbreviations: Anx = anxiety symptoms (HAM-A); Depr = depressive symptoms (IDS-SR); Psy = subclinical psychotic symptoms (PQ-16); Alc = alcohol use (AUDIT); Can = cannabis Use (CUDIT); Self = self-esteem (SER-SR); SoAn = social anxiety symptoms (SIAS); SoFu = psychosocial functioning (WSAS); Hedo = Hedonism Questionnaire; ObCo = obsessive-compulsive symptoms (Y-BOCS); Trau = post-traumatic stress symptoms (IES-R); MoS = alertness and motor speed (CRT); ViA = sustained visual attention and processing speed (RVP); FreR = verbal recognition memory-immediate (VRM); PIQ = premorbid IQ (NART); EpM = episodic memory and learning (PAL); FrFu = planning test (OTS); StrU = strategy use (SWM); Dela = verbal recognition memory- delayed (VRM); RuAc = rule acquisition and attentional set shifting, cognitive flexibility (IED); VeM = verbal memory (CVLT); VeF = verbal fluency (GIT).

# **Table S6.** Edge weights of the associations between cognitive domains and psychopathology domains^a^

| **Variable** | Anx | Depr | Psy | Alc | Can | Self | SoAn | SoFu | Hedo | ObCo | Trau | **Sum** |
| --- | --- | --- | --- | --- | --- | --- | --- | --- | --- | --- | --- | --- |
| MoS | -.008 | -.009 | -.031 | .021 | 0 | 0 | 0 | 0 | -.056 | 0 | 0 | **-.083** |
| ViA | 0 | 0 | -.033 | .023 | -.028 | 0 | 0 | 0 | 0 | 0 | -.045 | **-.083** |
| FreR | 0 | -.011 | -.001 | 0 | 0 | 0 | 0 | 0 | 0 | 0 | 0 | **-.012** |
| PIQ | 0 | 0 | -.05 | 0 | -.001 | 0 | 0 | 0 | 0 | 0 | -.002 | **-.053** |
| EpM | 0 | 0 | 0 | 0 | 0 | 0 | 0 | 0 | 0 | 0 | 0 | **0** |
| FrFu | 0 | 0 | 0 | 0 | 0 | 0 | 0 | 0 | 0 | -.006 | 0 | **-.006** |
| StrU | 0 | 0 | 0 | .05 | 0 | 0 | 0 | 0 | 0 | -.026 | -.017 | **.007** |
| Dela | 0 | -.037 | -.04 | 0 | 0 | 0 | 0 | 0 | 0 | 0 | 0 | **-.077** |
| RuAc | 0 | 0 | 0 | 0 | 0 | 0 | 0 | 0 | 0 | 0 | 0 | **0** |
| VeM | 0 | 0 | -.005 | 0 | -.028 | 0 | .014 | 0 | -.035 | .02 | 0 | **-.034** |
| VeF | 0 | 0 | 0 | .037 | -.037 | 0 | 0 | 0 | -.013 | .024 | 0 | **.011** |
| **Sum** | **-.008** | **-.057** | **-.16** | **.131** | **-.094** | **0** | **.014** | **0** | **-.104** | **.012** | **-.064** |  |

^a^Rows represent cognitive domains and columns indicate psychopathology domains. The final column and row represent sums. The table corresponds with Table S 3.

# **Table S7.** Results of the bootstrap analysis of the EGA clustering^a^

| **Dimensions** | **2** | **3** | **4** | **5** | **6** | **7** | **8** | **≥9** | **Median** | **SD** |
| --- | --- | --- | --- | --- | --- | --- | --- | --- | --- | --- |
| Main network (n= 1016) | 2 | 745 | 237 | 15 | 1 | - | - | - | 3 | .49 |
| High education (n= 538) | 2 | 90 | 299 | 293 | 142 | 58 | 52 | 64 | 5 | 1.75 |
| Low education (n= 431) | 5 | 415 | 437 | 116 | 25 | 2 | - | - | 4 | .78 |
| Substance use fused (n= 1016) | 178 | 640 | 165 | 17 | - | - | - | - | 3 | .64 |
| No zero substance use (n= 570) | 60 | 271 | 354 | 226 | 52 | 24 | 8 | 5 | 4 | 1.19 |
| Male patients (n= 478) | 78 | 477 | 279 | 126 | 32 | 8 | - | - | 3 | .97 |
| Female patients (n= 537) | - | 154 | 731 | 83 | 26 | 6 | - | - | 4 | .63 |
| No Anxiety Disorders (n= 956) | 1 | 635 | 341 | 23 | - | - | - | - | 3 | .53 |
| No Depressive Disorders (n= 905) | 1 | 723 | 194 | 67 | 14 | 1 | - | - | 3 | .68 |
| No Misphonia (n= 663) | - | 283 | 620 | 91 | 6 | - | - | - | 4 | .60 |
| No OCD (n= 839) | 11 | 891 | 94 | 4 | - | - | - | - | 3 | .34 |
| No SZ Disorders (n= 831) | 3 | 398 | 492 | 79 | 23 | 5 | - | - | 4 | .75 |
| No Other Disorders (n= 886) | 20 | 628 | 311 | 36 | 5 | - | - | - | 3 | .61 |
| Older patients (n= 519) | 21 | 217 | 344 | 253 | 107 | 52 | 6 | - | 4 | 1.18 |
| Younger patients (n= 497) | 10 | 373 | 382 | 167 | 48 | 16 | 4 | - | 4 | .99 |
| Without medication use (n= 392) | - | 34 | 149 | 213 | 171 | 135 | 99 | 199 | 6 | 2.35 |
| WIth medication use (n= 611) | 27 | 225 | 558 | 163 | 24 | 3 | - | - | 4 | 0.78 |

Abbreviation: SZ= Schizophrenia spectrum and other psychotic disorders, OCD= Obsessive-compulsive and related disorders, Misophonia= Impulse-control disorder NOS (misophonia).

^a^Table shows the number of bootstraps for each number of dimensions per condition and the median number of dimensions per condition. The maximum number of conditions is 17.

# **Table S8.** Results of the bootstrap analysis of the EGA clustering- Node placement^a^

| **Cluster** | **Nodes** |
| --- | --- |
| 1 | MoS, ViA, FrR, PIQ, EpM, FrF, StU, Del, RuA, VeM, VeF |
| 2 | Anx, Depr, Psy, Self, SoAn, SoFu, Hedo, ObCo, Trau |
| 3 | Alc, Can |

^a^Table that demonstrates to which cluster all the nodes in the network belonged in the median network as obtained by bootstrapping.

Abbreviations: Anx = anxiety symptoms (HAM-A); Depr = depressive symptoms (IDS-SR); Psy = subclinical psychotic symptoms (PQ-16); Alc = alcohol use (AUDIT); Can = cannabis Use (CUDIT); Self = self-esteem (SER-SR); SoAn = social anxiety symptoms (SIAS); SoFu = psychosocial functioning (WSAS); Hedo = Hedonie Questionnaire; ObCo = obsessive-compulsive symptoms (Y-BOCS); Trau = post-traumatic stress symptoms (IES-R); MoS = alertness and motor speed (CRT); ViA = sustained visual attention and processing speed (RVP); FreR = verbal recognition memory-immediate (VRM); PIQ = premorbid IQ (NART); EpM = episodic memory and learning (PAL); FrFu = planning test (OTS); StrU = strategy use (SWM); Dela = verbal recognition memory- delayed (VRM); RuAc = rule acquisition and attentional set shifting, cognitive flexibility (IED); VeM = verbal memory (CVLT); VeF = verbal fluency (GIT).

# **Figure S1.** Strength centrality measure of psychopathology and cognitive domains.


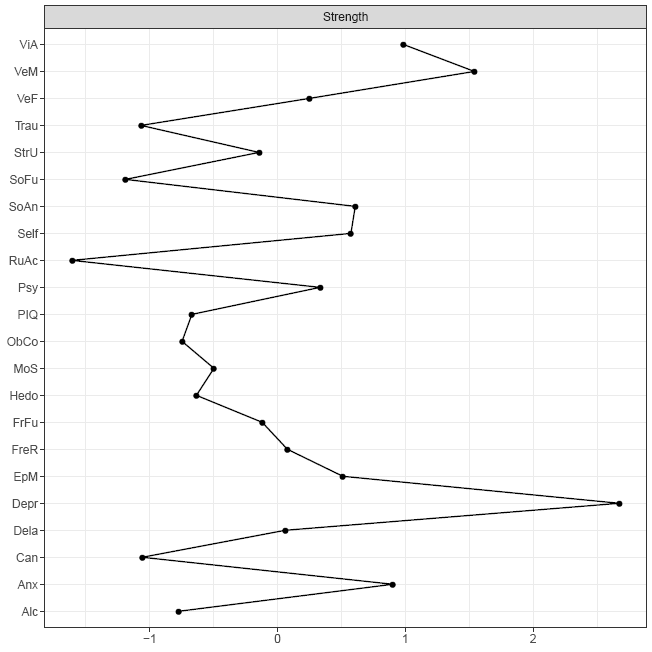


Strength indicates the degree of association of that node to its neighbors. See Figure S5 below regarding the stability of the centrality indices.

# **Figure S2.** Accuracy of the estimated edges of the network.


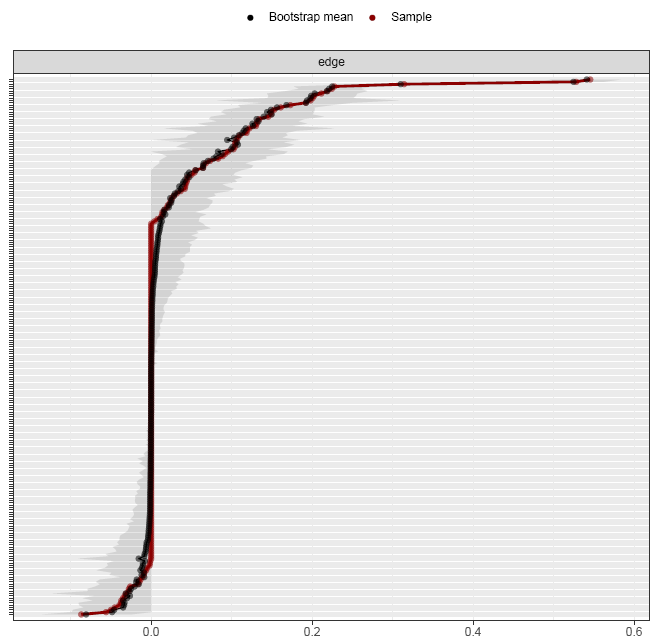


The x-axis shows the strength of the edge. The edges from the original network are shown in red and are arranged from most negative to most positive along the y-axis. The grey area represents confidence intervals based on the bootstrapped networks.

# **Figure S3.** Difference tests of edges in the network


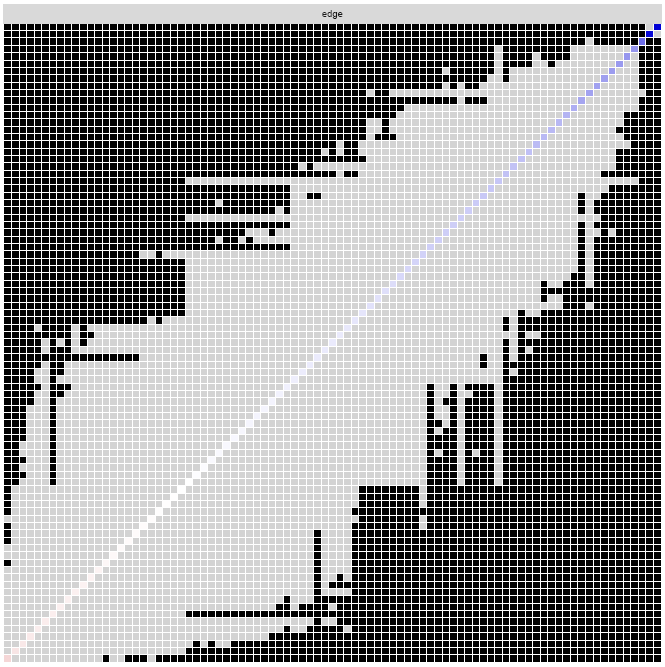


Rows and columns represent the different edges in the network, with edges ordered from most negative weight (left, bottom) to most positive (right, top). Black dots indicate that two edges differ from each other at the α = 0.05 level.

# **Figure S4.** Difference test of total strength per node in the network


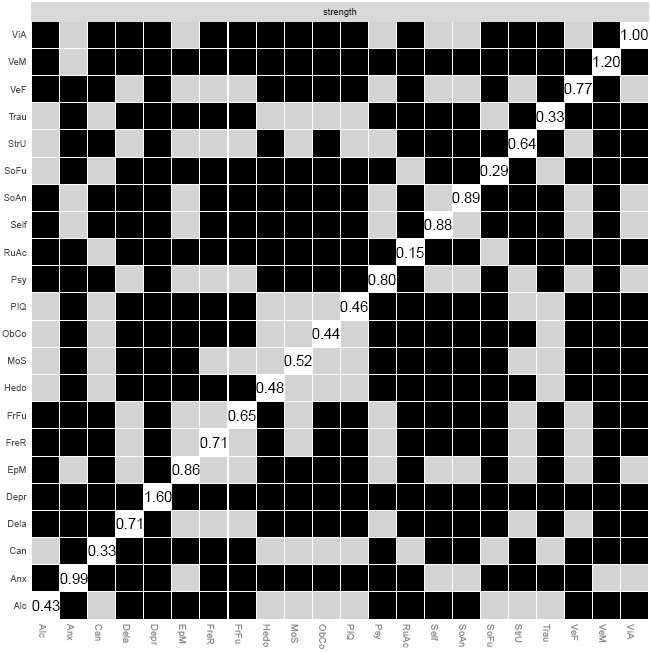


Black dots represent significant differences at the α = 0.05 level. The diagonal line contains the centrality index.

# **Figure S5.** Stability of centrality indices of the network after ‘dropping’ a part of the patients


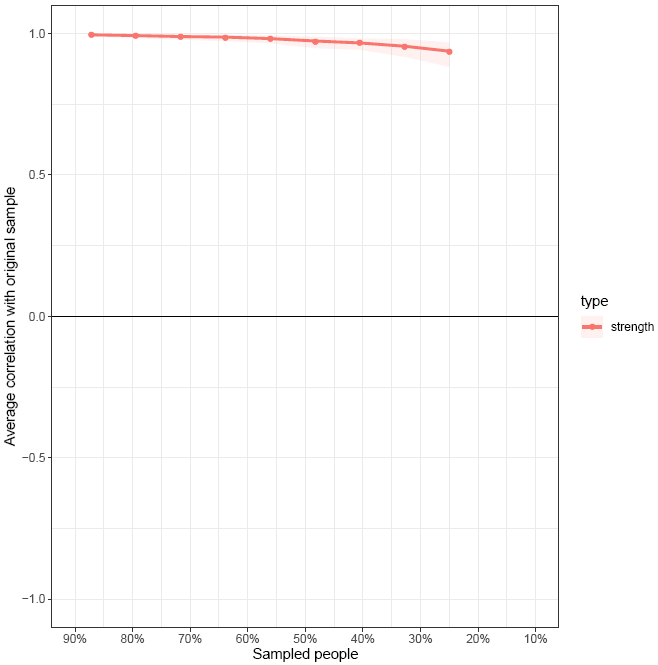


The x-axis shows the percentage of patients that was dropped. The y-axis shows the correlation of the centralities after dropping to the original centralities. The shaded areas indicate the 95% confidence interval. The centralities are considered stable as long as the 95% confidence interval does not reach 0.7.

# **Figure S6.** Networks for substance use fused and no zero substance use


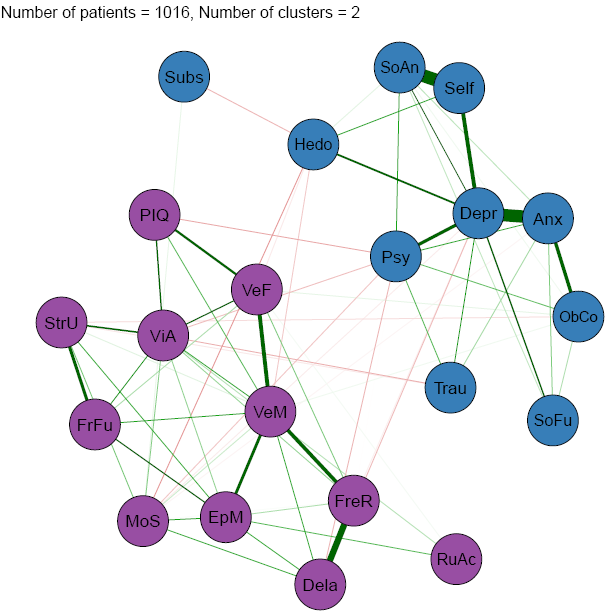

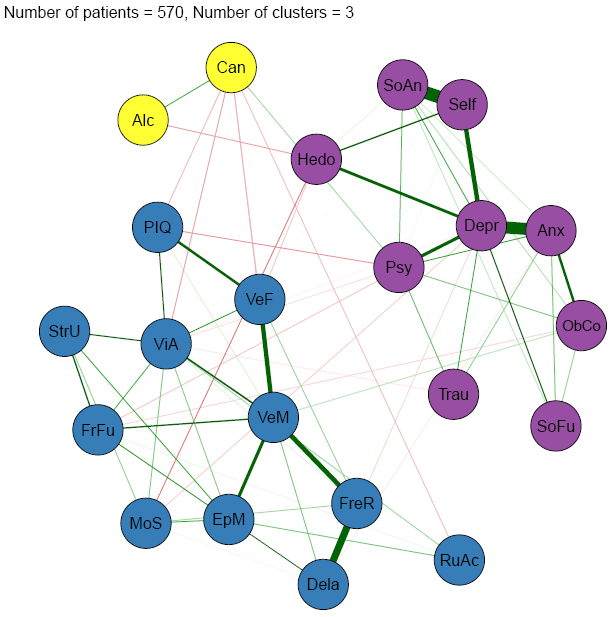


a

b

Networks made with the EBICglasso procedure (a and b) for the following conditions: substance use fused (a) and no zero substance use (b). Nodes represent the variables included in the network and edges indicate an association between two nodes. Green edges represent positive associations whereas red edges represent negative associations, and thickness of an edge represents the strength of association between two nodes. Colors represent cluster membership as determined by the EGA algorithm.

# **Figure S7.** Accuracy of the estimated edges of substance use control networks


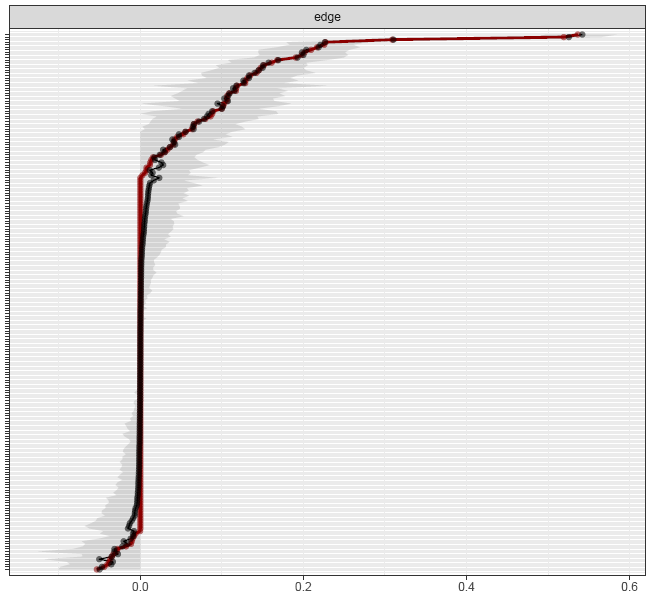

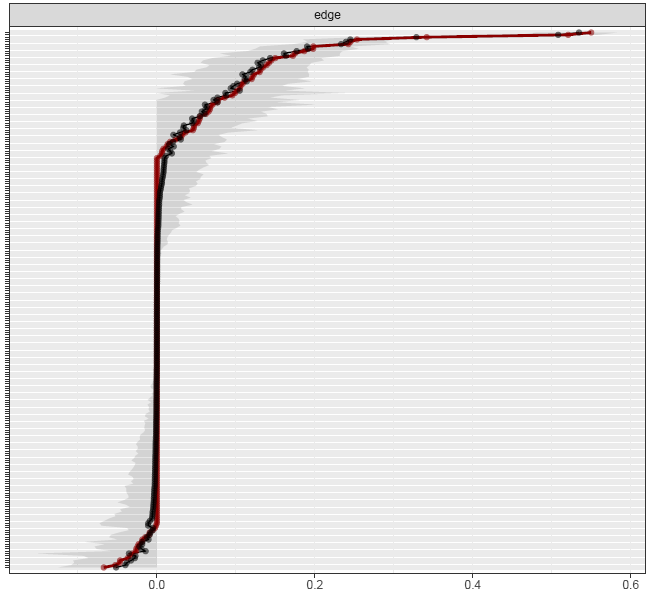


b

a

Networks: substance use fused (a) and no zero substance use (b). The x-axis shows the strength of the edge. The edges from the original network are shown in red and the bootstrapped means are shown in black. Edges are arranged from most negative to most positive along the y-axis. The grey area represents confidence intervals based on the bootstrapped networks.

# **Figure S8.** Networks of subsamples by demographics: education level, sex, and age


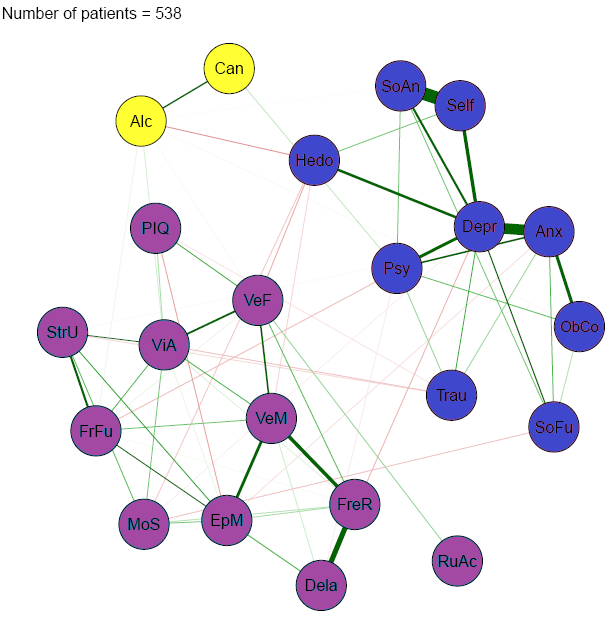

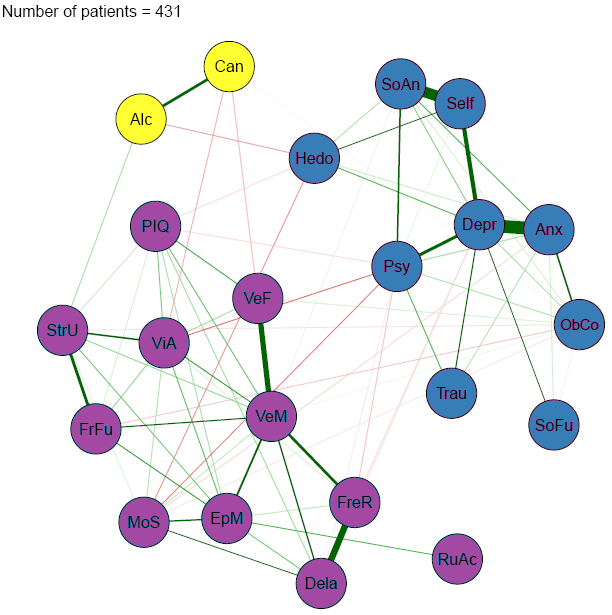


a

b


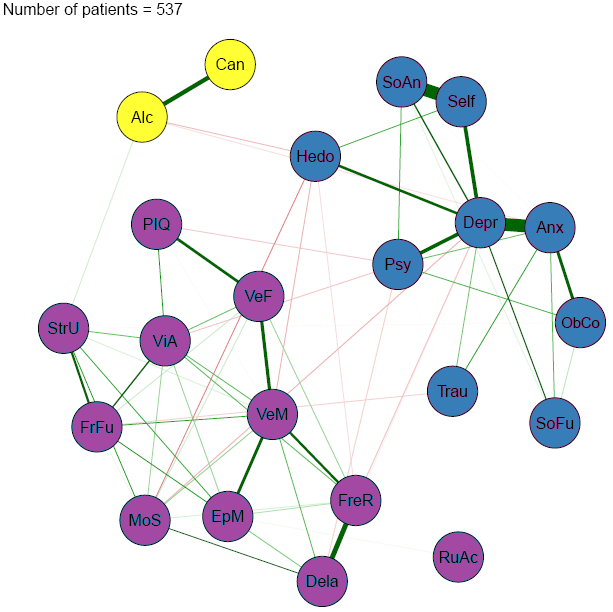

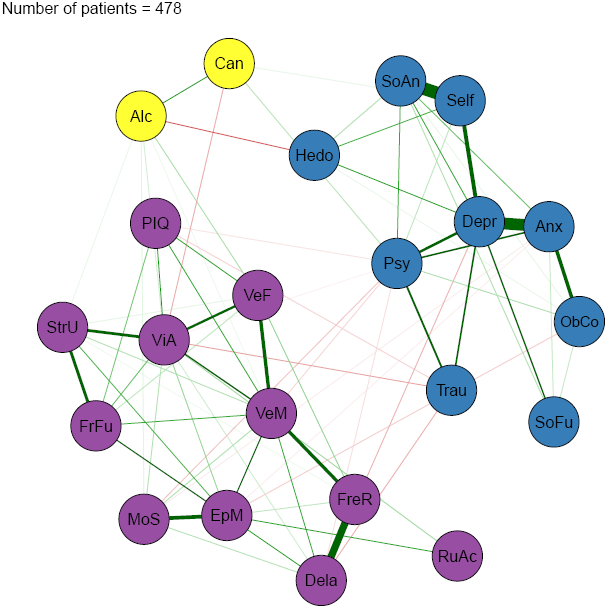


d

c


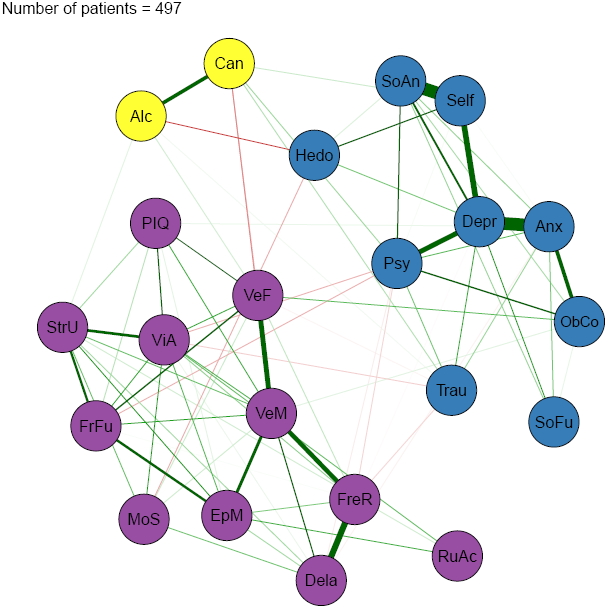

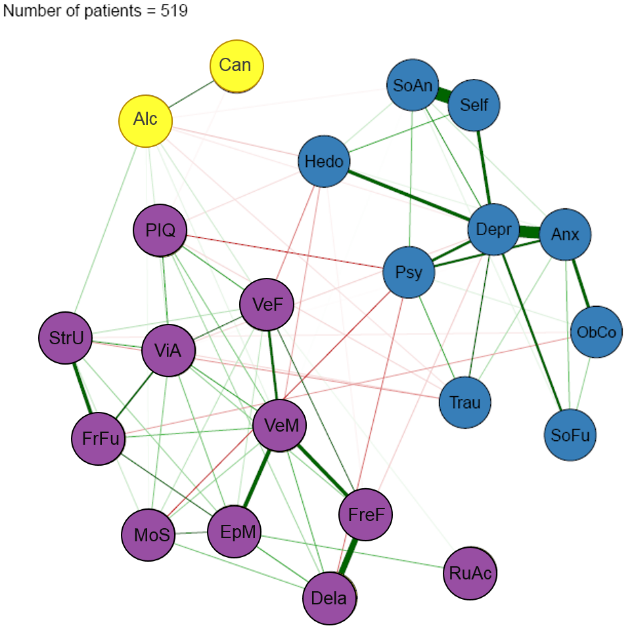


f

e

Network made with the EBICglasso procedure for the subsample of patients with high educational level (a), patients with low educational level (b), female patients (c), male patients (d), younger patients aged 14-30 years (e), and older patients aged 31-75 years (f). Nodes represent the variables included in the network and edges indicate an association between two nodes. Green edges represent positive associations whereas red edges represent negative associations, and thickness of an edge represents the strength of association between two nodes. Colors represent cluster membership as determined by the EGA algorithm.

# **Figure S9.** Accuracy of the estimated edges of demographic control networks


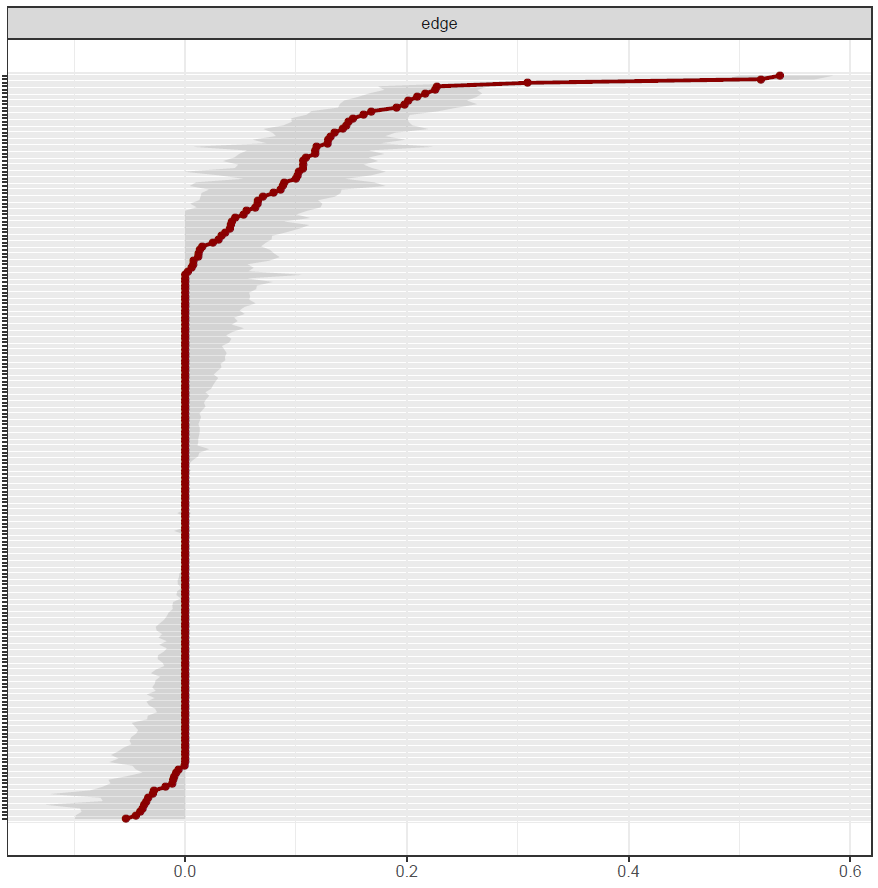

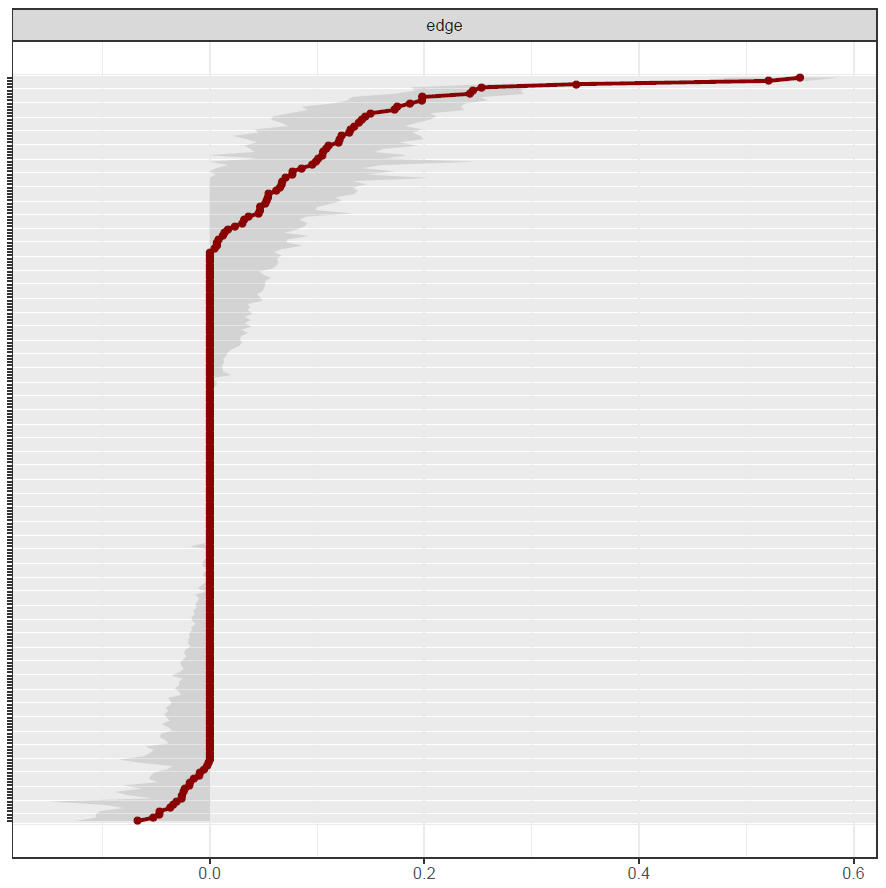

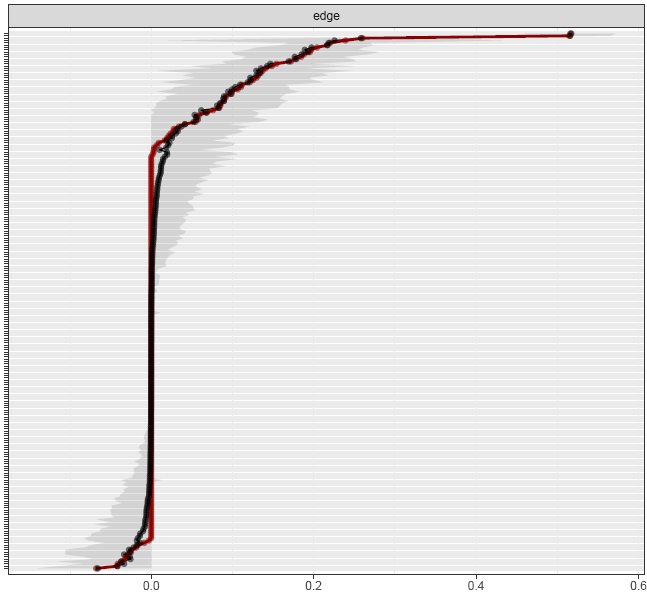

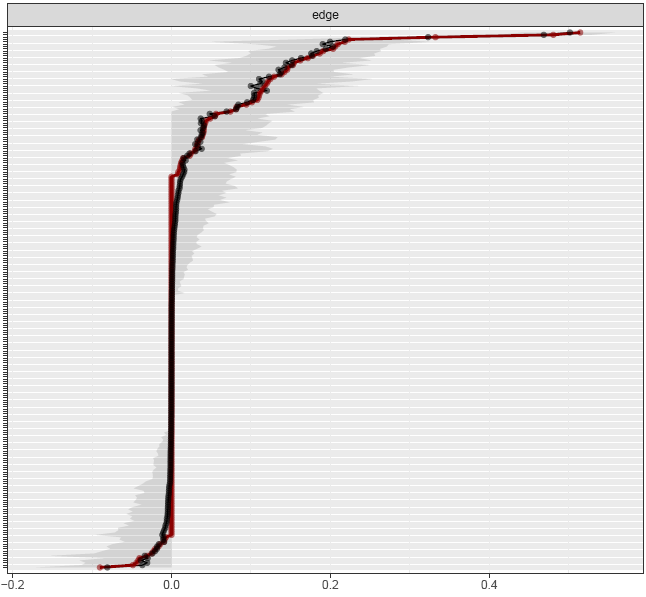


b

c

a

d


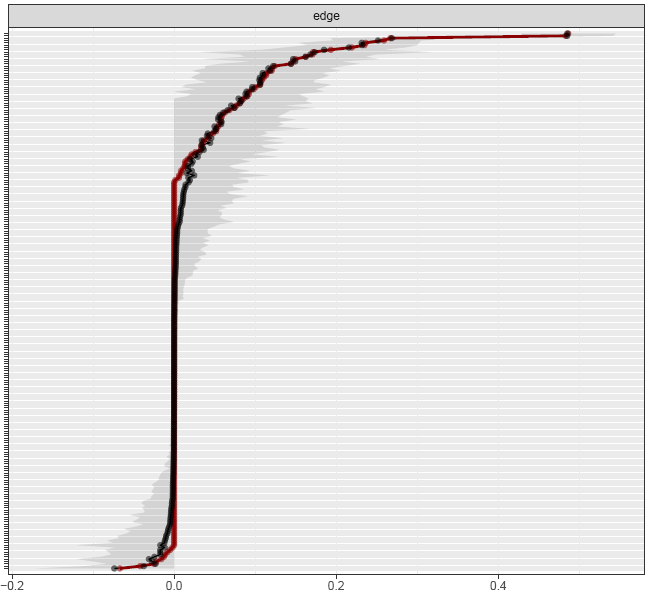

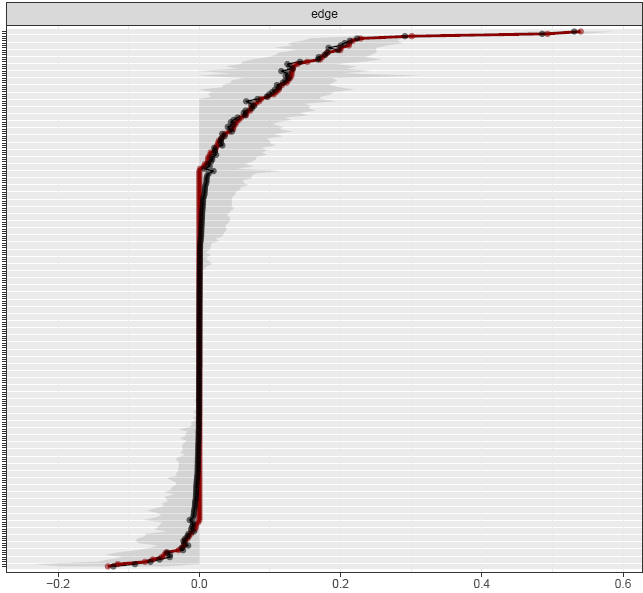


f

e

Networks: high education (a), low education (b), female patients (c), male patients (d), younger patients (e), and older patients (f). The x-axis shows the strength of the edge. The edges from the original network are shown in red and the bootstrapped means are shown in black. Edges are arranged from most negative to most positive along the y-axis. The grey area represents confidence intervals based on the bootstrapped networks.

# **Figure S10.** Networks of diagnostic subsamples


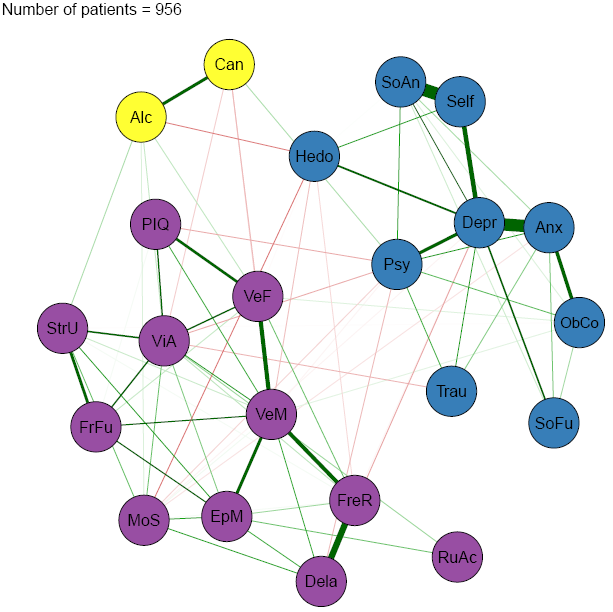

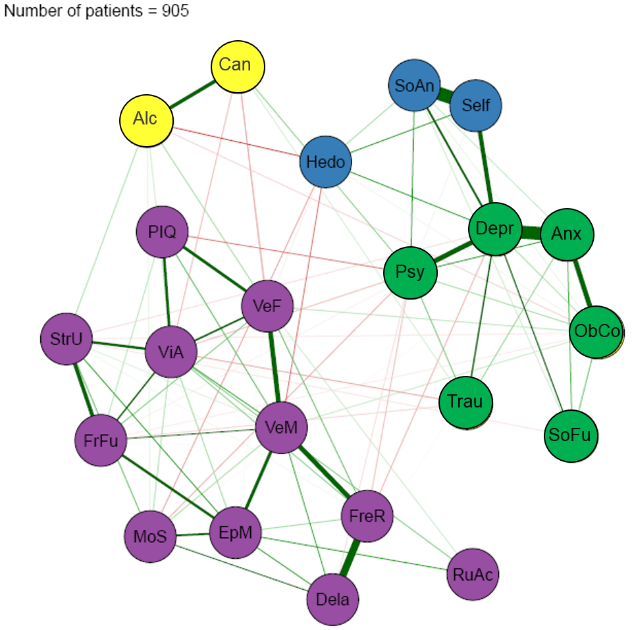


a

b


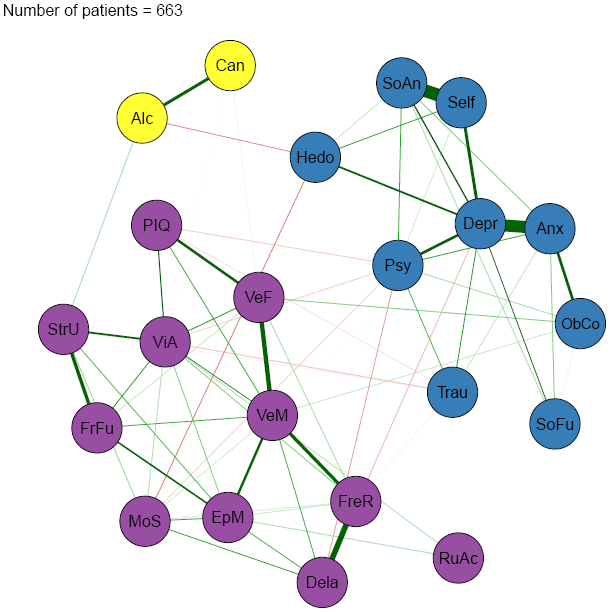

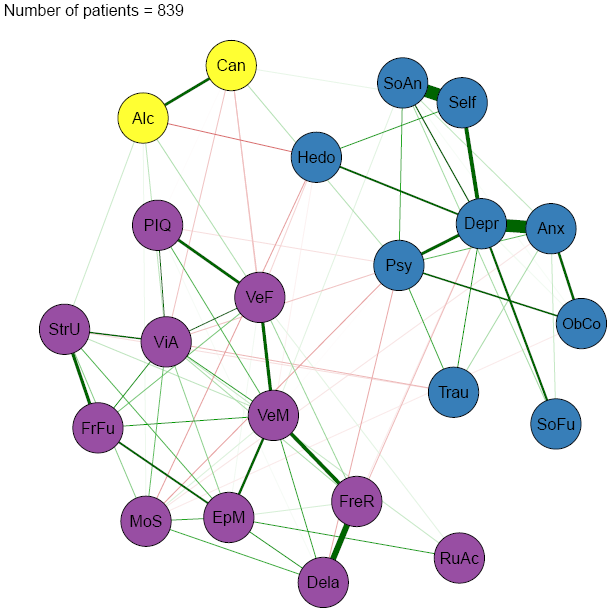


d

c


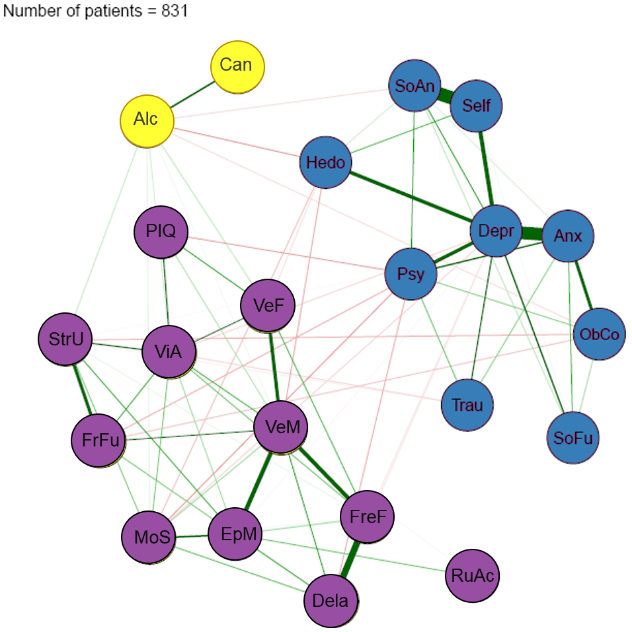

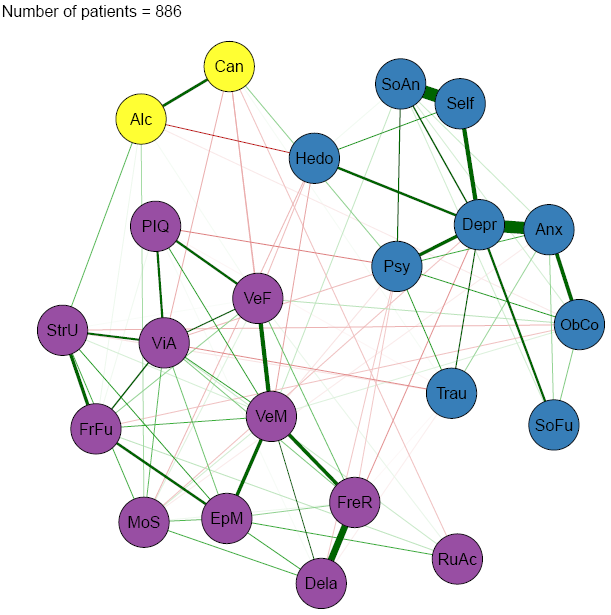


f

e

Network made with the EBICglasso procedure using the jackknife technique for the diagnostic categories subsamples without anxiety disorders (a), depressive disorders (b), misophonia (c), obsessive-compulsive and related disorders (d), schizophrenia spectrum and other psychotic disorders (e), and other disorders (f). Nodes represent the variables included in the network and edges indicate an association between two nodes. Green edges represent positive associations whereas red edges represent negative associations, and thickness of an edge represents the strength of association between two nodes. Colors represent cluster membership as determined by the EGA algorithm.

# **Figure S11.** Accuracy of the edges of diagnostic subsample control networks


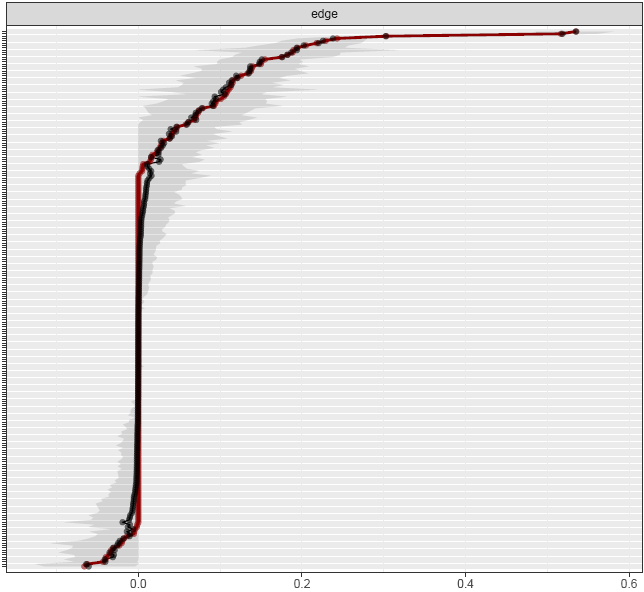

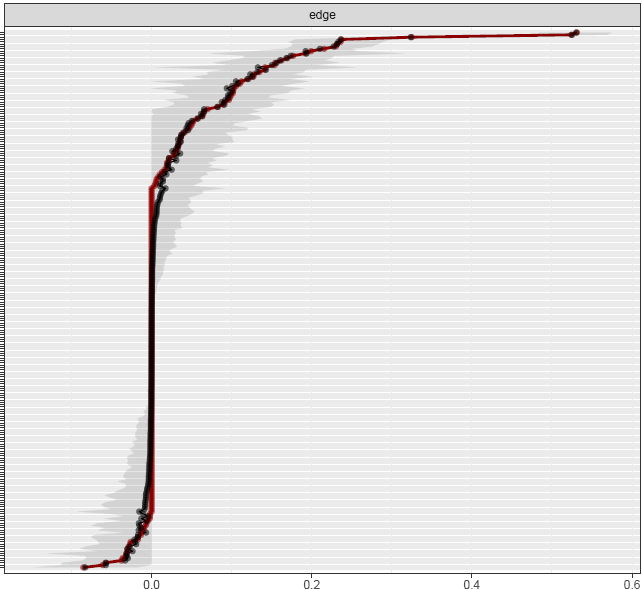

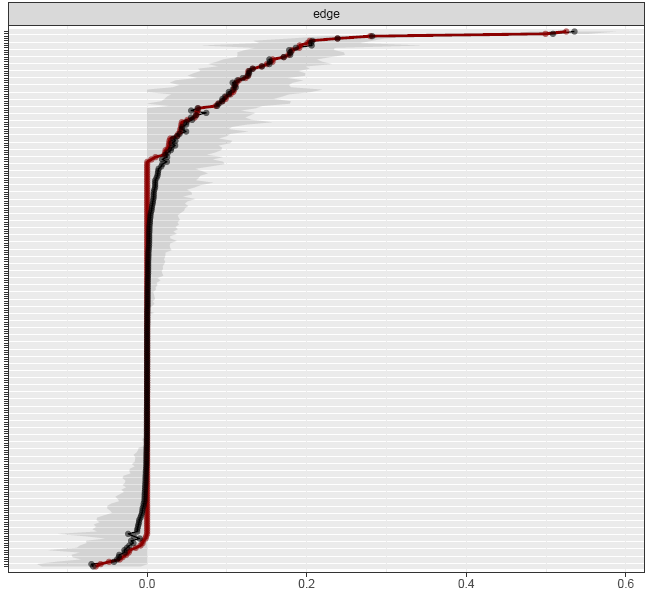

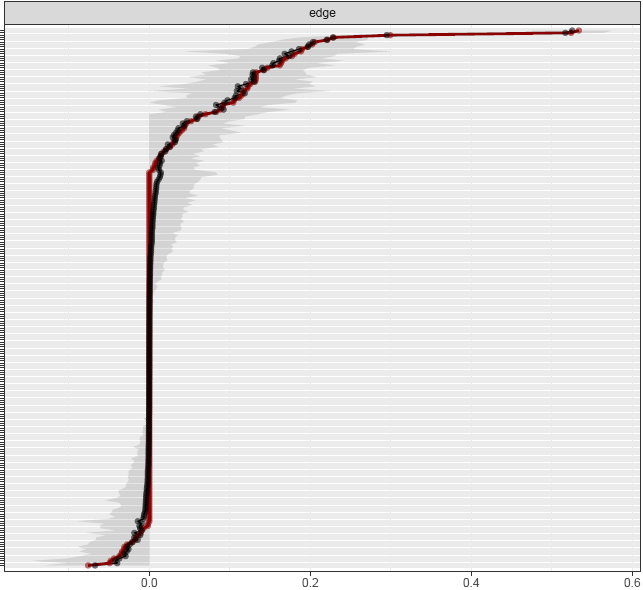


b

c

a

d


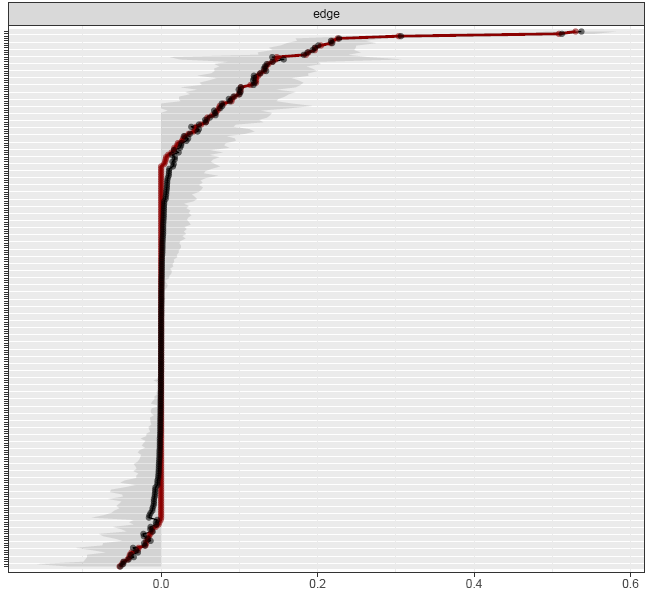

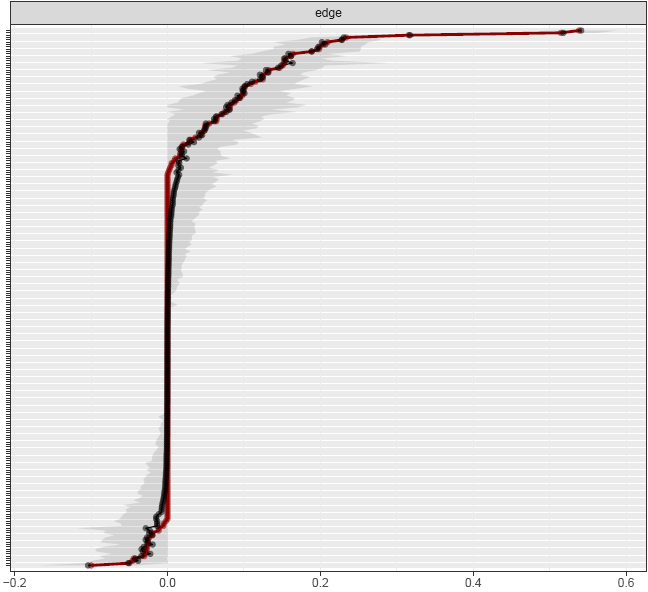


f

e

Networks: without anxiety disorders (a), depressive disorders (b), misophonia (c), obsessive-compulsive and related disorders (d), schizophrenia spectrum and other psychotic disorders (e), and other disorders (f).The x-axis shows the strength of the edge. The edges from the original network are shown in red and the bootstrapped means are shown in black. Edges are arranged from most negative to most positive along the y-axis. The grey area represents confidence intervals based on the bootstrapped networks.

#
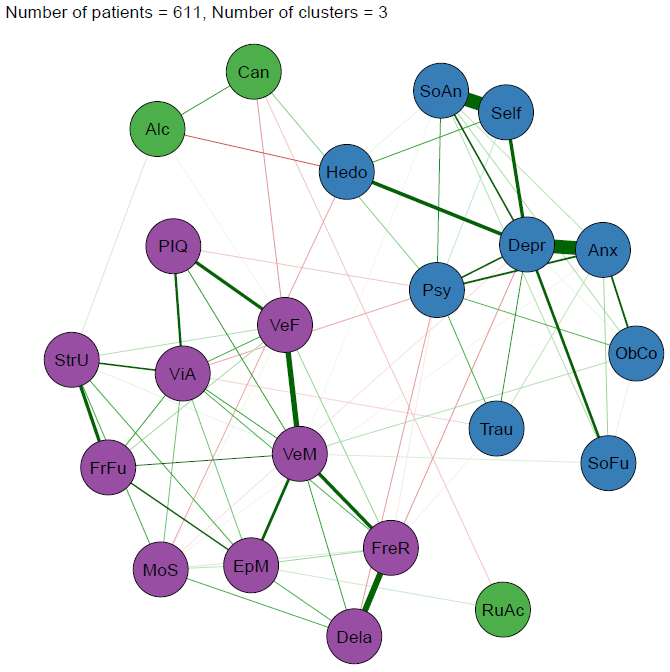

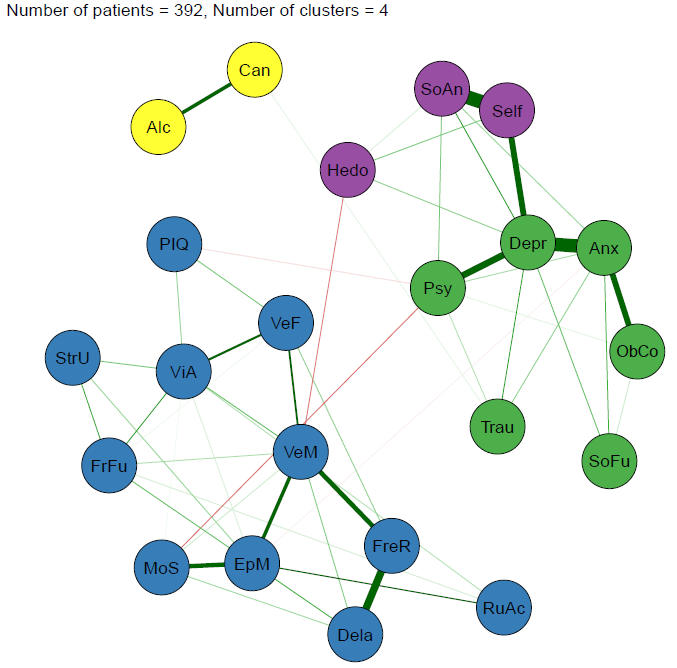
**Figure S12.** Networks of medication use subsamples

b

a

Network made with the EBICglasso procedure for the subsample of patients without medication use (a) and patients with medication use (b). Nodes represent the variables included in the network and edges indicate an association between two nodes. Green edges represent positive associations whereas red edges represent negative associations, and thickness of an edge represents the strength of association between two nodes. Colors represent cluster membership as determined by the EGA algorithm.

# **Figure S13.** Accuracy of the edges of medication use subsample control networks


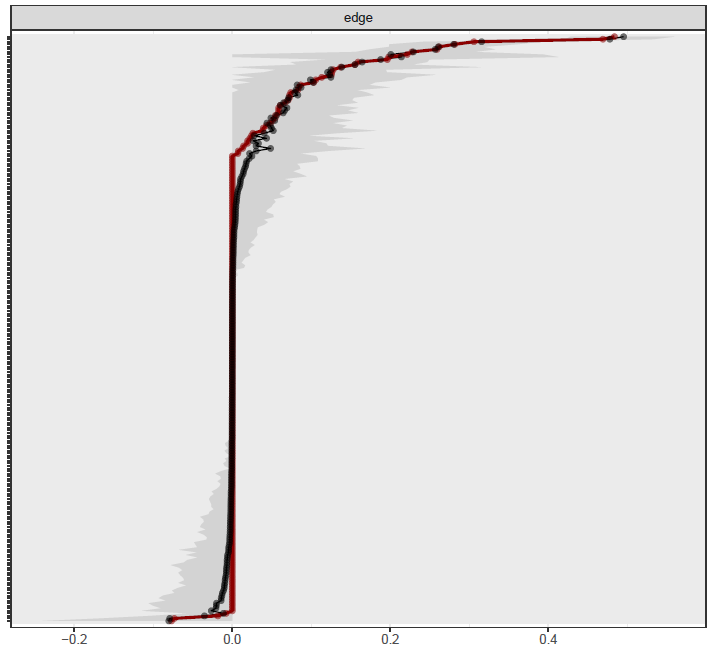

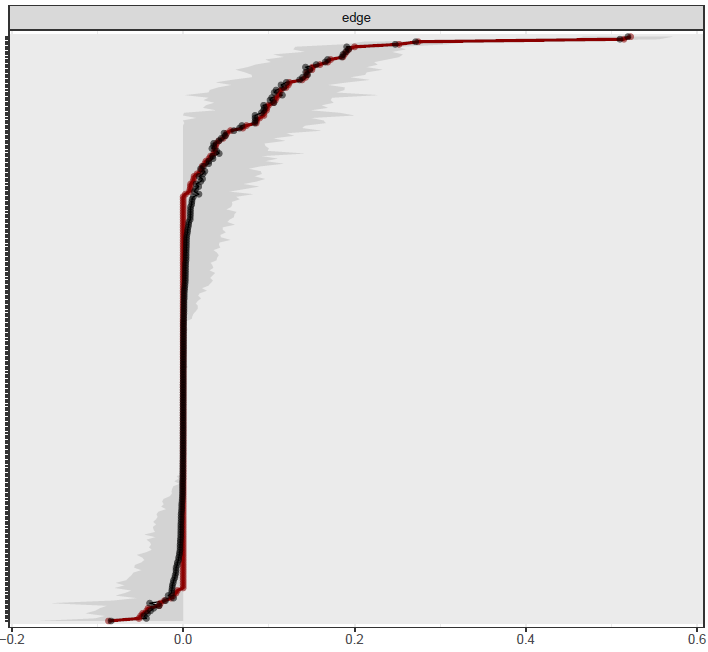


b

a

Networks: without medication use (a) and with medication use (b). The x-axis shows the strength of the edge. The edges from the original network are shown in red and the bootstrapped means are shown in black. Edges are arranged from most negative to most positive along the y-axis. The grey area represents confidence intervals based on the bootstrapped networks.

# **Figure S14.** Ising network of 690 patients using binarized data and the *IsingFit* package


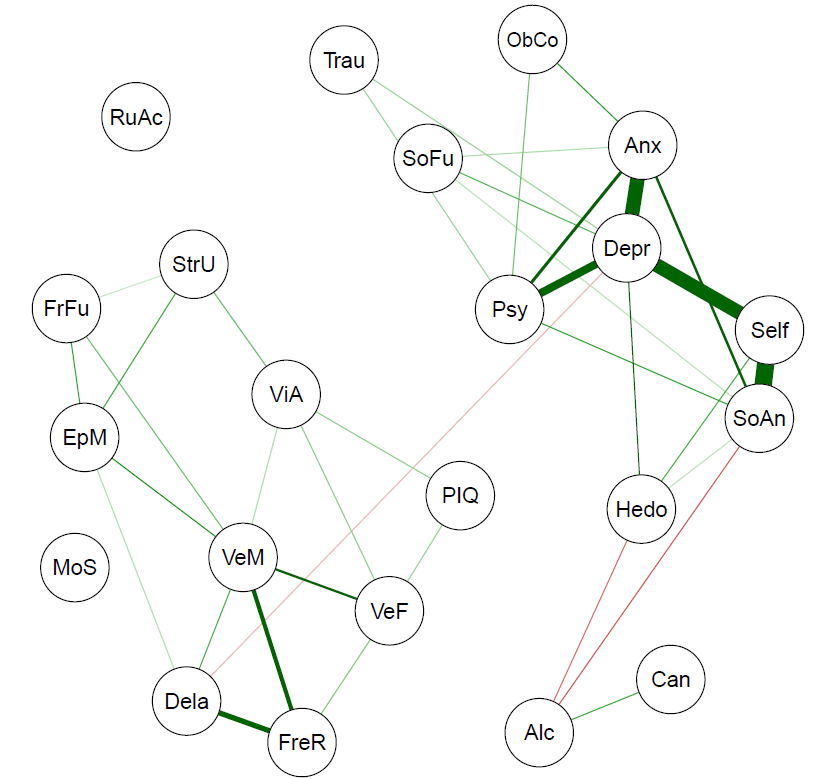


Nodes represent the variables included in the network and edges indicate an association between two nodes. Green edges represent positive associations whereas red edges represent negative associations, and thickness of an edge represents the strength of association between two nodes.

# **References**

Adamson, S. J., & Sellman, J. D. (2003). A prototype screening instrument for cannabis use disorder: the Cannabis Use Disorders Identification Test (CUDIT) in an alcohol‐dependent clinical sample. *Drug and alcohol review, 22*(3), 309-315. doi:https://doi.org/10.1080/0959523031000154454

Cambridge Cognition. (2018). CANTAB® [Cognitive assessment software].

Chen, J., & Chen, Z. (2008). Extended Bayesian information criteria for model selection with large model spaces. *Biometrika, 95*(3), 759-771. doi:https://doi.org/10.1093/biomet/asn034

Chernick, M. (2007). What Is Bootstrapping. *Bootstrap Methods: A Guide for Practitioners and Researchers*, 1-25.

Delis, D. C., Kramer, J., Kaplan, E., & Ober, B. A. (2000). *CVLT-II: California verbal learning test: adult version*: Psychological Corporation.

Efron, B., & Tibshirani, R. J. (1994). *An introduction to the bootstrap*: CRC press.

Epskamp, S., Borsboom, D., & Fried, E. I. (2018). Estimating psychological networks and their accuracy: A tutorial paper. *Behavior Research Methods, 50*(1), 195-212. doi:https://doi.org/10.3758/s13428-017-0862-1

Foygel, R., & Drton, M. (2010). *Extended Bayesian information criteria for Gaussian graphical models.* Paper presented at the Advances in Neural Information Processing Systems.

Friedman, J., Hastie, T., & Tibshirani, R. (2008). Sparse inverse covariance estimation with the graphical lasso. *Biostatistics, 9*(3), 432-441. doi:https://doi.org/10.1093/biostatistics/kxm045

Golino, H. F., & Epskamp, S. (2017). Exploratory graph analysis: A new approach for estimating the number of dimensions in psychological research. *PloS One, 12*(6), e0174035. doi:10.1371/journal.pone.0174035

Goodman, W., Price, L., Rasmussen, S., Mazure, C., Fleischmann, R., Hill, C., . . . Charney, D. (1989). Yale-brown obsessive compulsive scale (Y-BOCS): I. Development, use, and reliability. *Archives of General Psychiatry, 46*, 1006-1011. doi:10.1001/archpsyc.1989.01810110048007

Hamilton, M., Schutte, N., & Malouff, J. (1976). Hamilton anxiety scale (HAMA). *Sourcebook of Adult Assessment: Applied Clinical Psychology*, 154-157.

Ising, H. K., Veling, W., Loewy, R. L., Rietveld, M. W., Rietdijk, J., Dragt, S., . . . Linszen, D. H. (2012). The validity of the 16-item version of the Prodromal Questionnaire (PQ-16) to screen for ultra high risk of developing psychosis in the general help-seeking population. *Schizophrenia Bulletin, 38*(6), 1288-1296. doi:https://doi.org/10.1093/schbul/sbs068

Lecomte, T., Corbière, M., & Laisné, F. (2006). Investigating self-esteem in individuals with schizophrenia: relevance of the Self-Esteem Rating Scale-Short Form. *Psychiatry Research, 143*(1), 99-108. doi:https://doi.org/10.1016/j.psychres.2005.08.019

Luteijn, F., & Van der Ploeg, F. A. (1983). Groninger Intelligentie Test: Handleiding [Groninger Intelligence Test: Manual]. *Swets, Zeitlinger BV: Lisse*.

Mattick, R. P., & Clarke, J. C. (1998). Development and validation of measures of social phobia scrutiny fear and social interaction anxiety. *Behaviour Research and Therapy, 36*(4), 455-470.

Mundt, J. C., Marks, I. M., Shear, M. K., & Greist, J. M. (2002). The Work and Social Adjustment Scale: a simple measure of impairment in functioning. *British Journal of Psychiatry, 180*(5), 461-464. doi:https://doi.org/10.1192/bjp.180.5.461

Opsahl, T., Agneessens, F., & Skvoretz, J. (2010). Node centrality in weighted networks: Generalizing degree and shortest paths. *Social networks, 32*(3), 245-251.

Rombouts, R., & Van-Kuilenburg, C. J. (1988). Hedonie, de ontwikkeling van een vragenlijst [Development of a questionnaire designed to measure hedonism]. *Gedrag en Gezondheid, 16*, 117-123.

Rush, A. J., Giles, D. E., Schlesser, M. A., Fulton, C. L., Weissenburger, J., & Burns, C. (1986). The inventory for depressive symptomatology (IDS): preliminary findings. *Psychiatry Research, 18*(1), 65-87. doi:https://doi.org/10.1016/0165-1781(86)90060-0

Saunders, J. B., Aasland, O. G., Babor, T. F., De la Fuente, J. R., & Grant, M. (1993). Development of the alcohol use disorders identification test (AUDIT): WHO collaborative project on early detection of persons with harmful alcohol consumption‐II. *Addiction, 88*(6), 791-804. doi:https://doi.org/10.1111/j.1360-0443.1993.tb02093.x

Schmand, B., Bakker, D., Saan, R., & Louman, J. (1991). The Dutch Reading Test for Adults: a measure of premorbid intelligence level. *Tijdschrift voor Gerontologie en Geriatrie, 22*(1), 15-19.

Van Borkulo, C. D., Borsboom, D., Epskamp, S., Blanken, T. F., Boschloo, L., Schoevers, R. A., & Waldorp, L. J. (2014). A new method for constructing networks from binary data. *Scientific Reports, 4*, 5918.

Verhage, F. (1964). *Intelligentie en leeftijd: Onderzoek bij Nederlanders van twaalf tot zevenenzeventig jaar*: van Gorcum.

Weiss, D., Marmar, C., Wilson, J., & Keane, T. (1997). The Impact of Event Scale-Revised. In *Assessing Psychological Trauma and PTSD*. New York, NY, US: Guilford Press.
